# A Lie Bracket for the Momentum Kernel

**DOI:** 10.1007/s00220-023-04748-z

**Published:** 2023-06-30

**Authors:** Hadleigh Frost, Carlos R. Mafra, Lionel Mason

**Affiliations:** 1 The Mathematical Institute, University of Oxford, Andrew Wiles Building, ROQ, Woodstock Rd, Oxford, OX2 6GG UK; 2 Mathematical Sciences and STAG Research Centre, University of Southampton, Highfield, Southampton, SO17 1BJ UK

## Abstract

We prove results for the study of the double copy and tree-level colour/kinematics duality for tree-level scattering amplitudes using the properties of Lie polynomials. We show that the ‘*S*-map’ that was defined to simplify super-Yang–Mills multiparticle superfields is in fact a Lie bracket. A generalized KLT map from Lie polynomials to their dual is obtained by studying our new Lie bracket; the matrix elements of this map yield a recently proposed ‘generalized KLT matrix’, and this reduces to the usual KLT matrix when its entries are restricted to a basis. Using this, we give an algebraic proof for the cancellation of double poles in the KLT formula for gravity amplitudes. We further study Berends–Giele recursion for biadjoint scalar tree amplitudes that take values in Lie polynomials. Field theory amplitudes are obtained from these ‘Lie polynomial amplitudes’ using numerators characterized as homomorphisms from the free Lie algebra to kinematic data. Examples are presented for the biadjoint scalar, Yang–Mills theory and the nonlinear sigma model. That these theories satisfy the Bern–Carrasco–Johansson amplitude relations follows from the structural properties of Lie polynomial amplitudes that we prove.

## Introduction

The results in this paper show how Lie polynomials [1] and the combinatorics of words [2] are basic to the study of tree-level scattering amplitudes in field theory and string theory. We give self-contained proofs of the identities that underpin coloured amplitudes and the double copy at tree-level using simple properties of Lie polynomials. We will see that the cleanest description is in terms of the Berends–Giele multiparticle fields of biadjoint scalar theory, with values in Lie polynomials.

The free Lie algebra, ${\mathscr {L}}$, is the space of linear combinations of ‘Lie monomials’, which are nested commutators of ‘letters’; our letters will be taken to be the natural numbers ($1,2,3,\ldots$). ${\mathscr {L}}$ is a subspace of the space of linear combinations of ‘words’ formed from the natural numbers. The connection to gauge theory arises because there is a natural map from Lie monomials $\Gamma \in {\mathscr {L}}$ to the colour structures that appear in gauge theories, for any choice of gauge Lie algebra, and for any (single trace) gauge theory Lagrangian. Moreover, there is also a correspondence between Lie monomials $\Gamma \in {\mathscr {L}}$ and labelled binary trees with a given root.

The double copy starts by expressing Yang-Mills tree amplitudes in the form [3]

1.1 $$\begin{aligned} A= \sum _\Gamma \frac{N_\Gamma c_\Gamma }{s_\Gamma }\,. \end{aligned}$$

Here $\Gamma$ denotes trivalent graphs, $s_\Gamma$ denotes the product of denominator propagator factors associated to the graph, and $c_\Gamma$ denotes the corresponding colour factor.^1^ The numerators $N_\Gamma$ are functions of momenta and gluon polarization data. These are said to be ‘BCJ numerators’ if they satisfy colour-kinematics duality, which means that they satisfy

$$\begin{aligned} N_{\Gamma }+N_{\Gamma '}+N_{\Gamma ''}=0, \end{aligned}$$

whenever $\Gamma +\Gamma '+\Gamma ''= 0$. In other words, the $N_\Gamma$ are ‘BCJ numerators’ if $\Gamma \mapsto N_\Gamma$ is a homomorphism from ${\mathscr {L}}$ to the space of functions of the kinematic data.^2^ Such numerators exist for Yang-Mills, and the key example of the double copy is that replacing $c_\Gamma$ in (1.1) by another copy of $N_\Gamma$ yields gravity amplitudes [4]. BCJ numerators are known for many coloured theories and can be used to obtain the tree amplitudes of any theory known to participate in the double copy. This includes gauge and gravity theories and their relatives, such as brane theories, with and without supersymmetry, as well as tree-level string amplitudes; see [5] for an up-to-date review of progress and references to the literature. The most basic example is to replace $N_\Gamma$ in (1.1) by $c_\Gamma$. This yields the amplitudes of the biadjoint scalar theory, which is the backbone of the double copy.

Lie polynomials are ubiquitous in the auxiliary structures that are used to study amplitudes, and with hindsight can be seen in the multiparticle vertex operators in conventional string theory [6], in the geometry of the space of Mandelstam variables [7], and in the CHY formulae and ambitwistor strings [8, 9], where a prominent role is played by $\mathcal {M}_{0,n}$, the moduli space of *n*-points on the Riemann sphere [10, 11]. However, our aim here is to prove basic results directly using only the Lie polynomial structure.

The following sections summarize our results.

### Berends–Giele recursion and planar binary trees

In Sect. 2, we review the properties of the space of Lie polynomials, ${\mathscr {L}}$, and its dual ${\mathscr {L}}^*$. Elements of the dual, ${\mathscr {L}}^*$, can be expressed as ‘words modulo shuffles’. For a Lie monomial $\Gamma \in {\mathscr {L}}$ and a word *P*, the duality pairing is denoted $(P,\Gamma )$. There is a correspondence between Lie monomials $\Gamma \in {\mathscr {L}}$ (up to sign) and binary trees, i.e., trivalent rooted tree graphs [12]. If *P* is a word such that $(P,\Gamma )=1$, then *P* defines a planar embedding of the tree associated to $\Gamma$.

Section 3 reviews the Berends–Giele recursion relations for biadjoint scalar theory. For $P\in {\mathscr {L}}^*$, we reduce this problem to studying the recursion (as in [13])

1.2 $$\begin{aligned} b(P)=\frac{1}{s_P} \sum _{XY=P} [b(X),b(Y)]\,, \qquad b(i) = i \end{aligned}$$

where $s_P$ is the Mandelstam variable associated to the word *P*. The *b*(*P*) are valued in the space of Lie polynomials, ${\mathscr {L}}$. Moreover, the relation (1.2) is solved by

1.3 $$\begin{aligned} b(P)=\sum _\Gamma \frac{(P,\Gamma ) \Gamma }{s_\Gamma }, \end{aligned}$$

where the sum is over Lie monomials $\Gamma$, defined up to sign.

The *b*(*P*) naturally define what we call *Lie polynomial amplitudes*. These are obtained by removing the off-shell external propagator to obtain colour ordered partial amplitudes *m*(*Pn*) valued in Lie polynomials:

1.4 $$\begin{aligned} m(Pn):=\lim _{s_P\rightarrow 0} s_P b(P). \end{aligned}$$

The pairing of (1.4) with an ordering gives the double colour ordered partial amplitudes of the biadjoint theory $m(Pn,Qn):=(Q,m(Pn))$ [14].

For a given gauge theory, we interpret BCJ numerators $N_\Gamma$ as given by homomorphism, *N*, from the free Lie algebra to functions of their kinematic data, as in [10]. The existence of such a homomorphism is special to those gauge theories that participate in the double copy, and we write down examples for NLSM and SYM theories in § 7. The amplitudes are obtained by acting on *m*(*Pn*) with *N*. They satisfy the Kleiss Kuijf (KK) relations and the Bern Carrasco Johansson (BCJ) relations because of the basic results that we prove about the ${\mathscr {L}}$-valued *b*(*P*).

### BCJ amplitude relations from a new Lie bracket

It was argued in [6–15] that BCJ amplitude relations could be expressed using the map defined in [6] that was there called the ‘*S*-map’. We will show that this map defines a Lie bracket in the dual space of Lie polynomials. We will call this Lie bracket the *S*-*bracket* and denote it with braces: $\{\,,\}$.

We prove that the BCJ amplitude relations of [6–15] follow from the basic identity

1.5 $$\begin{aligned} b(\{P,Q\})= \left[ b(P),b(Q)\right] , \end{aligned}$$

which generalizes the off-shell BCJ relations of [16]. Thus *b* maps the $\{,\}$-bracket to the standard Lie bracket. We also show that $b: P\mapsto b(P)$ is an invertible map, and that $\{,\}$ is the pullback of [, ] under this map. In particular $\{,\}$ is a Lie bracket.

The BCJ relations for amplitudes are a consequence of (1.5), which implies that $b(\{P,Q\})$ does not have pole in $1/s_{PQ}$, and hence that

$$\begin{aligned} m(\{P,Q\},n)=0, \end{aligned}$$

in the limit as $s_{PQ}\rightarrow 0$.

### The KLT inner product and its generalized matrix

The Kawai–Lewellen-Tye (KLT) matrix [17–19] relates Yang-Mills partial amplitudes to gravity amplitudes. It arises in a natural way from the *S*-bracket. If $\Gamma$ is a Lie monomial, let $\{\Gamma \}$ be obtained by replacing every pair of brackets [, ] with an *S*-bracket $\{,\}$. This is well defined because the *S*-bracket is Lie. We use this to define a KLT map:

$$\begin{aligned} S: \Gamma \mapsto \{\Gamma \}, \end{aligned}$$

which is valued in ${\mathscr {L}}^*$. Using the duality pairing between ${\mathscr {L}}$ and ${\mathscr {L}}^*$, the KLT map defines a symmetric bilinear form on ${\mathscr {L}}$: $S(\Gamma _1,\Gamma _2):=(\{\Gamma _1\}, \Gamma _2)$. The conventional KLT matrix is recovered by evaluating the matrix elements of this map in a basis. In particular, the ‘generalized KLT matrix’ of [13] is given by

1.6 $$\begin{aligned} S(P,Q)= (\{\ell [P]\},\ell [Q])\,, \end{aligned}$$

where $\ell$ denotes the complete left bracketings:

1.7 $$\begin{aligned} \ell [123\ldots n]:=[\ldots [1,2]\ldots n]\,. \end{aligned}$$

Cachazo, He and Yuan [8] showed that biadjoint scalar amplitudes are in some sense the inverse to the KLT matrix, see [20–22]. Using the Berends–Giele formula for biadjoint amplitudes, [14], this statement precisely follows from the basic statement that the maps *b* and *S* are inverses of each other, which is our main result.

### Numerators and cobrackets

In Sect. 8 we show that the “contact term map” defined in [23] is the Lie co-bracket dual to $\{~,~\}$; it gives rise to a *Lie co-algebra* structure on ${\mathscr {L}}^*$. In the context of pure spinor superstrings, [23] the contact-term map encodes the BRST variations of local multiparticle superfield numerators satisfying generalized Jacobi identities [6, 24, 25]. These BRST variations play a central role in the recent developments in the explicit calculation of superstring amplitudes, from tree-level to 3-loops.

Moreover, in Sect. 7 we study BCJ numerators. Our approach shows that BCJ-like numerators always exist, and are given by $N_\Gamma = B(\{\Gamma \})$, if *B*(*P*) are the Berends–Giele currents of the theory. However, there is no guarantee that these $N_\Gamma$ are local, except in special cases. We review known numerators including for the non-linear sigma model (NLSM), where we give a conjecture that has since been proved elsewhere [26]. We also study the numerators for super–Yang–Mills (sYM), Z-theory and the open superstring.

## Review of Lie Polynomials, Combinatorics on Words and Colour Factors

Let *W* be the vector space of linear combinations of words over the natural numbers. The free Lie algebra ${\mathscr {L}}$ is the subspace of *W* linearly spanned by Lie monomials, $\Gamma$. A Lie monomial is a complete bracketing of a word, such as such as

2.1 $$\begin{aligned} \Gamma = 123 - 132 - 231 + 321 = [1,[2,3]]. \end{aligned}$$

The left- and right-bracketings are surjective maps from *W* onto ${\mathscr {L}}$ given by

2.2 $$\begin{aligned} \begin{aligned} \ell [123\ldots n]&:= [[[1,2],3],\ldots ,n],\\ r[123\ldots n]&:= [1,[2,[3,\ldots ,[n-1,n]\ldots ]]]. \end{aligned} \end{aligned}$$

For a letter *i* and a word *P*,

2.3 $$\begin{aligned} \ell [Pi] = [\ell [P],i] \end{aligned}$$

(and similarly $r[iP] = [i,r[P]]$). This inductively implies Baker’s identity [1]

2.4 $$\begin{aligned} \ell [P\ell [Q]] = [\ell (P),\ell [Q]]. \end{aligned}$$

Write |*P*| for the length of a word *P*. If $\overline{P}$ denotes the reverse of *P*, then the so-called ‘antipode’ can be defined as

2.5 $$\begin{aligned} \alpha (P):= (-1)^{|P|} \overline{P}. \end{aligned}$$

The antipode relates $\ell [P]$ and *r*[*P*] by

2.6 $$\begin{aligned} \ell [P] = - r[\alpha (P)]. \end{aligned}$$

### ${\mathscr {L}}$ and its dual ${\mathscr {L}}^*$

This section recalls the duality pairing between ${\mathscr {L}}$ and its dual, ${\mathscr {L}}^*$, which is central to the results of the paper. For words $P,Q \in W$, write (*P*, *Q*) for the trivial inner product on *W*:

2.7 $$\begin{aligned} (P,Q):= {\left\{ \begin{array}{ll} 1 &{} \text { if } P=Q;\\ 0 &{} \text { otherwise}. \end{array}\right. } \end{aligned}$$

The shuffle product on *W*,  , is inductively defined by2.8for letters *i*, *j*, and words *P*, *Q*. The base case is . The expression  is sometimes referred to as the sum over *ordered permutations* of *P* and *Q*, preserving the ordering of the letters of *P* and of *Q*. Ree’s theorem characterizes ${\mathscr {L}}$ in terms of the shuffle product:

Theorem (Ree) [27]. $\Gamma \in W$ is a Lie polynomial iff  for all nonempty $P,Q\in W$.

Write $Sh \subset W$ for the subspace spanned by all *proper shuffles*, , with *P*, *Q* nonempty. Ree’s theorem implies that ${\mathscr {L}}$ is the orthogonal subspace of *Sh*, with respect to ( ,  ). Thus the dual vector space to ${\mathscr {L}}$ (with respect to ( ,  )) is given by the vector space quotient,

2.9 $$\begin{aligned} {\mathscr {L}}^* = W / Sh. \end{aligned}$$

Elements of ${\mathscr {L}}^*$ are equivalence classes, $P+Sh$, for some $P\in W$. If two expressions in *W*, *P* and *Q*, belong to the same equivalence class, write $P\sim Q$. If $P\sim Q$, then there exist some words $A_i,B_i$ and coefficients $c_{i}$ so that2.10On account of the ambiguity in how to represent elements of ${\mathscr {L}}^*$ and ${\mathscr {L}}$, it is useful to find bases.

A word *P* is *Lyndon* if it is smaller in the dictionary ordering than any of its suffixes: *P* is Lyndon if $P=QR$ for nonempty *Q* and *R*, then $P<R$ in the dictionary ordering. The Lyndon words, *P*, give a basis of ${\mathscr {L}}^*$ [1]. Dually, the set of Lie monomials, $\ell [P]$, for Lyndon words *P*, is a basis of ${\mathscr {L}}$. These two bases are dual because, for two Lyndon words *P* and *Q*, the smallest letter must come first in both words. But, for any letter *i*,

2.11 $$\begin{aligned} (iP,\ell [iQ]) = (iP,iQ) = (P,Q), \end{aligned}$$

because the only term in the word expansion of $\ell [iQ]$ that has *i* at the beginning is *iQ*.

### Appearance in gauge theory

The algebra recalled above is ubiquitous in gauge theory because of the colour factors. In this context, it will be helpful to write $W_n \subset W$ for the words in *W* that have no repeated letters, and only involve letters $1,2,\ldots ,n$. Likewise write ${\mathscr {L}}_n = {\mathscr {L}}\cap W_n$ for the Lie monomials restricted to letters $1,2,\ldots ,n$. Its dual is ${\mathscr {L}}_n^* = W_n / (Sh \cap W_n)$.

Fix *n* elements of a Lie algebra: $t^a_i \in {\mathfrak {g}}$, for $i=1,\ldots ,n$. For any Lie monomial $\Gamma \in {\mathscr {L}}_{n-1}$, let $\textbf{t}(\Gamma )$ be obtained by writing $\Gamma$ as a nested bracketing of $1,\ldots ,n-1$, and replacing *i* with $t_i$ and [ ,  ] with the Lie bracket of ${\mathfrak {g}}$. This defines a linear map

2.12 $$\begin{aligned} \textbf{t}: {\mathscr {L}}_{n-1} \rightarrow {\mathfrak {g}}. \end{aligned}$$

If $\textrm{tr}$ is the invariant inner product on ${\mathfrak {g}}$, then for every Lie monomial $\Gamma \in {\mathscr {L}}_{n-1}$, the associated *colour factor* is

2.13 $$\begin{aligned} c_\Gamma = \textrm{tr}(\textbf{t}(\Gamma )t_n). \end{aligned}$$

The replacement $\Gamma \mapsto c_\Gamma$ also defines a homomorphism out of ${\mathscr {L}}_{n-1}$.

**Fig. 1:**
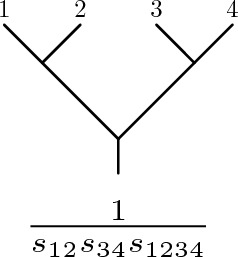
The product of propagators ${1\over s_\Gamma }$ and the planar binary tree associated to the Lie monomial [[1, 2], [3, 4]] according to the definition (2.17)

The colour factor $c_\Gamma$ arises in cubic scalar theory as the colour factor for a specific cubic Feynman graph, that we write as $T_\Gamma$. Regard $T_\Gamma$ as a rooted binary tree, with root at *n*. Then $T_\Gamma$ is defined inductively as follows. $T_{[1,2]}$ is the tree with two external legs, 1 and 2, connected by one vertex to the root. If $\Gamma = [\Gamma ',\Gamma '']$, then $T_\Gamma$ is the tree formed by connecting (or ‘grafting’) the roots of $T_{\Gamma '}$ and $T_{\Gamma ''}$ to make a new vertex. Every pair of brackets in $\Gamma$ corresponds to a vertex in $T_\Gamma$. An example is shown in Fig. 1. The trees $T_\Gamma$ and $T_{-\Gamma }$ are the same. So there is a 1:1 correspondence between Lie monomials *up to sign*, $\pm \Gamma$, and binary trees, *T*.

In massless theories, the contribution of a graph $T_\Gamma$ to the amplitude is a function of external momenta, $k_i^\mu$, $i=1,\ldots ,n$, with $k_i\cdot k_i = 0$. Write $s_{ij}$ for the Mandelstam variable

2.14 $$\begin{aligned} s_{ij}:= 2k_i\cdot k_j. \end{aligned}$$

For every subset $I\subset {\mathbb {N}}$ with at least two elements, write

2.15 $$\begin{aligned} k_I^\mu := \sum _{i\in I} k_i^\mu , \end{aligned}$$

and

2.16 $$\begin{aligned} s_I:= k_I^2 = \sum _{\{i,j\}\subset I} s_{ij}. \end{aligned}$$

Take $\Gamma$ and $T_\Gamma$ as above. When written as a nested bracket expression, each pair of brackets in $\Gamma$ defines a subset of $\{1,\ldots ,n-1\}$. If *I* is a subset that appears like this, write $I\in \Gamma$, and define

2.17 $$\begin{aligned} s_\Gamma := \prod _{I\in \Gamma } s_I. \end{aligned}$$

The inverse, $\frac{1}{s_\Gamma }$, is then the *product of propagators* of the tree graph, $T_\Gamma$, associated to $\Gamma$, including a propagator for the root of $T_\Gamma$.

Given this, it will be useful to write ${\mathcal {M}}$ for the Laurent ring in the variables $s_I$, subject to the relation (2.16). Clearly the propogator factors $1/s_\Gamma$ belong to ${\mathcal {M}}$.

### Identities for amplitudes

The duality between ${\mathscr {L}}_n$ and ${\mathscr {L}}_n^*$ leads to helpful identities that we collect in this section. A Lyndon basis for ${\mathscr {L}}_n^*$ is given by the words of the form 1*P*, that begin with 1. This basis is dual to the basis of ${\mathscr {L}}_n$ given by the corresponding Lie monomials $\ell [1P]$. Given that these are dual bases, we have the following basis expansion in ${\mathscr {L}}_n^*$,

2.18 $$\begin{aligned} P \sim \sum _Q (P, \ell [1Q]) 1Q \end{aligned}$$

where the sum is over all permutations, *Q*, of $23\ldots n$. For $iP\in W_n$ and some letter *i*, it can be checked that2.19This follows from (2.3). Substituting this formula for $\ell [iP]$ into (2.18) implies that,2.20for $XiY\in W_n$ is a permutation of $12\ldots n$, and some distinguished letter *i*.^3^ We will later see that this implies the Kleiss-Kuijf (KK) relations among partial amplitudes. Setting *Y* to be empty in (2.20) gives

2.22 $$\begin{aligned} P\sim -(-1)^{|P|}{\bar{P}}, \end{aligned}$$

for any word *P*.

We also have the dual basis expansion in ${\mathscr {L}}_n$. For $\Gamma \in {\mathscr {L}}_n$,

2.23 $$\begin{aligned} \Gamma = \sum _Q ( 1Q,\Gamma )\,\ell [1Q], \end{aligned}$$

where the sum is over permutations of $23\ldots n-1$. For example, this gives

2.24 $$\begin{aligned} \left[ [1,2],[3,4]\right] = \ell [1234] - \ell [1243], \end{aligned}$$

which follows also from the Jacobi identity.

Finally, we will need to use the *adjoints* of $\ell$ and *r*, which we write as $\ell ^*$ and $r^*$:

2.25 $$\begin{aligned} (\ell ^*(P),Q) = (P,\ell (Q)),\qquad (r^*(P),Q) = (P,r(Q)). \end{aligned}$$

It follows from (2.3) that the adjoints can be computed recursively as

2.26 $$\begin{aligned} \begin{aligned} \ell ^*(123 \ldots n)&= \ell ^*(123 \ldots n-1)n - \ell ^*(23 \ldots n)1,\qquad \ell ^*(i):= i,\\ r^*(123 \ldots n)&= 1r^*(23 \ldots n) - n r^*(123 \ldots n{-}1),\qquad r^*(i):= i,\\\end{aligned} \end{aligned}$$

Likewise, (2.19) implies that2.27Note that $\ell ^*$ and $r^*$ are well-defined on ${\mathscr {L}}^*$ as  for nonempty *P*, *Q*. This follows from , which vanishes by Ree’s theorem.

## Berends–Giele Recursion and Lie Polynomials

Berends–Giele (BG) is a recursive method to compute tree-level scattering amplitudes. [31] This section formulas Berends–Giele recursion in terms of fields with values in Lie polynomials. This is similar to the ‘perturbiner’ method of [15, 28, 32, 33], and will allow us to make full use of the properties of ${\mathscr {L}}_n$ and ${\mathscr {L}}_n^*$ reviewed in Sect. 2.^4^

### Berends–Giele recursion for biadjoint scalar theories

Consider a biadjoint scalar field $\Phi$ with values in the tensor product of two Lie algebras ${\mathfrak {g}}\otimes {\tilde{{\mathfrak {g}}}}$. Let these have structure constants $f^{abc}$ and ${\tilde{f}} ^{{\tilde{a}} {\tilde{b}} {\tilde{c}}}$ and invariant inner products for which we take an orthonormal basis. Then the Lagrangian is

3.1 $$\begin{aligned} {{\mathcal {L}}}_{BS} = \frac{1}{2} \nabla _\mu \Phi _{a{\tilde{a}}} \nabla ^\mu \Phi ^{a{\tilde{a}}} + \frac{1}{3!} f_{abc}{\tilde{f}}_{{\tilde{a}} {\tilde{b}} {\tilde{c}}} \Phi ^{a{\tilde{a}}}\Phi ^{b{\tilde{b}}}\Phi ^{c\tilde{c}}\,, \end{aligned}$$

where $\mu =1,\ldots ,d$ is a space-time index and *a*, *b* ${\tilde{a}}, {\tilde{b}}$ are Lie algebra indices. The field equations are

3.2 $$\begin{aligned} \Box \Phi _{a{\tilde{a}}}= \frac{1}{2} f_{abc}{\tilde{f}}_{{\tilde{a}} {\tilde{b}} {\tilde{c}}} \Phi ^{b{\tilde{b}}}\Phi ^{c{\tilde{c}}}\,. \end{aligned}$$

Our aim is to solve this field equation perturbatively. We will do this by solving a closely related problem. Let $\phi (x)$ be a field with values in ${\mathscr {L}}_n\otimes {\mathscr {L}}_n$ and subject to the field equaiton

3.3 $$\begin{aligned} \Box \phi = \frac{1}{2} [\![ \phi ,\phi ]\!] \,, \end{aligned}$$

where $[\![~,~]\!]$ is the symmetric bracket:

3.4 $$\begin{aligned}{}[\![\Gamma _1\otimes {{\tilde{\Gamma }}}_1,\Gamma _2\otimes {{\tilde{\Gamma }}}_2]\!]:=\left[ \Gamma _1,\Gamma _2\right] \otimes \left[ {{\tilde{\Gamma }}}_1,{{\tilde{\Gamma }}}_2\right] \,. \end{aligned}$$

For some null momenta $k_1,\ldots ,k_n$, the field

3.5 $$\begin{aligned} \phi _1=\sum _{j=1}^n e^{ik_j\cdot x}\, j\otimes j\, \in {\mathscr {L}}\otimes {\mathscr {L}}\,, \end{aligned}$$

gives a homogenous solution to (3.3). We use this to seed a recursive solution of (3.3).

Write ${\mathscr {L}}_{\le k}$ for the subspace of ${\mathscr {L}}_n$ spanned by Lie monomials $\Gamma$ with length $|\Gamma |\le k$. Given a solution to (3.3) with values in ${\mathscr {L}}_{\le k}$, consider the field

3.6 $$\begin{aligned} \phi _{k}= \phi _1+ \textrm{proj}_{\le k}\Box ^{-1} \frac{1}{2} [\![ \phi _{k-1},\phi _{k-1}]\!] \,, \end{aligned}$$

where $\textrm{proj}_{\le k}$ denotes the projection onto ${\mathscr {L}}_{\le k}$. Let us iteratively applying (3.6), starting with $\phi _1$. At each step in this recursion, the coefficient of a word *P* in $\Phi _k$ has *x* dependence $e^{ik_P\cdot x}$. So the inverse wave operator, $\Box ^{-1}$, acts on such a term to give $1/k_P^2=1/s_P$. When $k=n$, we have

3.7 $$\begin{aligned} \phi _{n}= \phi _1+ \Box ^{-1} \frac{1}{2} [\![ \phi _{n-1},\phi _{n-1}]\!] \,, \end{aligned}$$

which is a solution to (3.3), with values in ${\mathscr {L}}_n\otimes {\mathscr {L}}_n$. By construction, $\phi _n$ is symmetric in its two Lie polynomials factors. It follows that, for a word $P\in W_n$, pairing with the left or right factor of $\phi _n$ gives the same result: $(P,\phi _n) = (\phi _n,P)$. So write

3.8 $$\begin{aligned} \phi (P):= (P,\phi _n) = (\phi _n,P). \end{aligned}$$

The *x* dependence is given by $e^{ik_P\cdot x}$. So the Fourier transform of $\phi (P)$ to momentum space is just

3.9 $$\begin{aligned} b(P)= e^{-i k_P\cdot x} \Phi (P), \end{aligned}$$

which plays the same role in our context as a Berends–Giele ‘current’.

**Proposition** [13]. The Berends–Giele ‘currents’ satisfy

3.10 $$\begin{aligned} b(P) = {1\over s_P}\sum _{XY=P}[b(X),b(Y)], \qquad b(i) = i\,, \end{aligned}$$

where the sum is over all deconcatenations, $P=XY$, of *P*.

#### Proof

Consider the identity, for $\Gamma _1,\Gamma _2\in {\mathscr {L}}_n$,

3.11 $$\begin{aligned}{} & {} (P, \left[ \Gamma _{1},\Gamma _{2}\right] ) \left[ \Gamma _{1,}\Gamma _{2}\right] \nonumber \\{} & {} =\sum _{XY=P} \left[ (X,\Gamma _{1})\Gamma _{1}, (Y,\Gamma _{2})\Gamma _{2}\right] + \left[ (X,\Gamma _{2})\Gamma _{2}, (Y,\Gamma _{1})\Gamma _{1}\right] . \end{aligned}$$

This implies that

3.12 $$\begin{aligned} \frac{1}{2} [\![ \phi ,\phi ]\!](P) = \sum _{XY=P}[ \phi (X),\phi (Y)], \end{aligned}$$

and the result then follows by pairing (3.6) with *P*. $\square$

Moreover, since *b*(*P*) takes values in ${\mathscr {L}}_n$, Ree’s theorem implies that3.13for nonempty *R*, *S*. Thus $b: P \mapsto b(P)$ defines a homomorphism

3.14 $$\begin{aligned} b: {\mathscr {L}}_n^* \rightarrow {\mathscr {L}}_n \otimes {\mathcal {M}}, \end{aligned}$$

where ${\mathcal {M}}$ is the Laurent ring of Mandelstam variables.

The field theory $n+1$-particle amplitude can be obtained from *b*(*P*) by removing the last propagator, $1/s_{12\ldots n}$. Imposing momentum conservation, $s_{12\ldots n}\rightarrow 0$. So define the ‘${\mathscr {L}}_n$-valued amplitude’ by

3.15 $$\begin{aligned} m(P,n+1)=\lim _{s_{P}\rightarrow 0} s_{P}\, b(P), \end{aligned}$$

for a word $$P\in {\mathcal {L}}_{n-1}^*$$.

### The tree diagram expansion of *b*(*P*)

The recursion relation (3.10) can be solved explicitly, to recover the usual Feynman diagram expansion. Write $s_\Gamma$ for the product of variables $s_I$ defined in (2.17). Then we claim that

3.16 $$\begin{aligned} b(P) = \sum _\Gamma \frac{(P,\Gamma )\Gamma }{s_\Gamma }, \end{aligned}$$

where the sum is over $\Gamma \in {\mathscr {L}}_n$.

#### Lemma

The formula in (3.16) satisfies (3.10).

#### Proof

For any Lie monomial $\Gamma$ there are $\Gamma _1$ and $\Gamma _2$ so that $\Gamma =[\Gamma _1,\Gamma _2]$, and these monomials are unique up to sign. So, for a fixed Lie monomial $\Gamma$,

3.17 $$\begin{aligned} \frac{(P,\Gamma )\Gamma }{s_\Gamma }= \frac{(X,\Gamma _1)(Y,\Gamma _2) \left[ \Gamma _1,\Gamma _2\right] }{s_P s_{\Gamma _1}s_{\Gamma _2}} - \frac{(Y,\Gamma _1)(X,\Gamma _2) \left[ \Gamma _1,\Gamma _2\right] }{s_P s_{\Gamma _1}s_{\Gamma _2}}, \end{aligned}$$

where $P=XY$ and $|X|=|\Gamma _1|$, $|Y|=|\Gamma _2|$. Summing over all Lie monomials (up to sign), $\Gamma _1$ and $\Gamma _2$, that have length strictly smaller than |*P*| gives (3.10). $\square$

The first few examples of *b*(*P*) are:

3.18 $$\begin{aligned} \begin{aligned} b(12)&= {[1,2]\over s_{12}},\\ b(123)&= {[[1,2],3]\over s_{12}s_{123}} + {[1,[2,3]]\over s_{23}s_{123}},\\ b(1234)&= {[ [ [ 1, 2 ], 3 ], 4 ] \over s_{12} s_{123} s_{1234}} + {[ [ 1, [ 2, 3 ] ], 4 ] \over s_{123} s_{1234} s_{23}}\\ {}&+ {[ [ 1, 2 ], [ 3, 4 ] ] \over s_{12} s_{1234} s_{34}} + {[ 1, [ [ 2, 3 ], 4 ] ] \over s_{1234} s_{23} s_{234}} + {[ 1, [ 2, [ 3, 4 ] ] ] \over s_{1234} s_{234} s_{34}}. \end{aligned} \end{aligned}$$

**Fig. 2:**
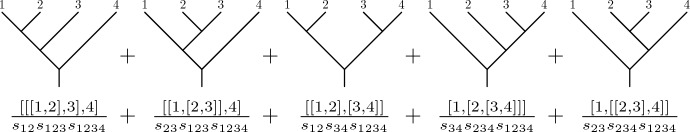
The Catalan expansion *b*(1234) from (3.10). Viewed as cubic graphs and removing the overall propagator $1/s_{1234}$, they correspond to the expansion of a color-ordered five-point tree amplitude *A*(12345) [13]. Note the leg 5 does not enter in the Lie elements in the numerators and that the root is unlabelled. By labelling the root and assigning leg 5 to the Catalan expansion of *b*(5) one recovers the free Lie algebra correspondence (3.21) for the case $n=5$

See Fig. 2 for this last example. Since *b*(*P*) is valued in ${\mathscr {L}}_n$, it can be expanded in a basis using (2.23), to get

3.19 $$\begin{aligned} b(P) = \sum _R b(P|iR)\ell [iR], \end{aligned}$$

where *i* is the smallest letter in *P*, and where

3.20 $$\begin{aligned} b(P|Q) = (b(P),\ell [Q]). \end{aligned}$$

The usual biadjoint scalar partial tree amplitude is then given by [14]

3.21 $$\begin{aligned} m(Pn,Qn)=: \lim _{s_{P}\rightarrow 0}s_{P} b(P|Q) = (Q,m(Pn)). \end{aligned}$$

We will see in Sect. 7 how *b*(*P*) can be dressed with BCJ numerators to give multiparticle fields and amplitudes for other gauge theories.

## A New Lie Bracket for Tree-Level Scattering Amplitude Relations

This section introduces the *S*-bracket, $\{~,~\}$, and shows that it is a Lie bracket. The *S*-bracket is then used to define the ‘generalized KLT matrix’ and to prove the identities conjectured in [13].

### The *S*-bracket

A bilinear map was introduced in [6–15] (where it was called the ‘S map’) to express the BCJ relations for super-Yang–Mills amplitudes from its action on Berends–Giele currents $M_P$ from [34]. It was abstracted to a map acting on words in [13] and the off-shell BCJ relations for *b*(*P*) was conjectured, but no general proof was given. Here we will see that this map defines a Lie bracket on ${\mathscr {L}}^*$, the ‘S bracket’, and this will lead to a new formulation of the fundamental BCJ relations.

#### Definition

(*S* bracket). Define a multilinear pairing $\{~,~\}: {\mathcal {L}}^*\otimes {\mathcal {L}}^* \rightarrow {\mathcal {L}}^*$ by [13]

4.1 $$\begin{aligned} \{P,Q\}:= r^*(P)\star \ell ^*(Q), \end{aligned}$$

where $r^*$ and $\ell ^*$ are defined in (2.26) and

4.2 $$\begin{aligned} Ai\star jB:= s_{ij}\, AijB \end{aligned}$$

for words *A*, *B* and letters *i*, *j*.

This definition implies that the *S*-bracket can be recursively computed using

4.3 $$\begin{aligned} \begin{aligned} \{iAj,B\}&=i\{Aj,B\}-j\{iA,B\},\\\{B,iAj\}&=\{B,iA\}j-\{B,Aj\}i,\\\{i,j\}&= s_{ij}\,ij \end{aligned} \end{aligned}$$

which follows from (2.27). Altneratively, for $P,Q\in {\mathscr {L}}^*$, equation (2.27) also gives an explicit closed formula: [6]4.4For example,

4.5 $$\begin{aligned} \begin{aligned} \{1,2\}&=s_{12}12, \\\{1,23\}&= s_{12} 123 - s_{13} 132,\\\{12,34\}&= s_{23}1234 - s_{24}1243 - s_{13} 2134 + s_{14}2143. \end{aligned} \end{aligned}$$

Given that the adjoints $r^*$ and $\ell ^*$ annihilate proper shuffles, the definition (4.1) manifestly satisfies .

The *S*-bracket is antisymmetric. Indeed, by (4.4),4.6because, in ${\mathscr {L}}^*$, $X\sim -(-1)^X{\overline{X}}$. It follows that,

4.7 $$\begin{aligned} \{P,Q\}\sim -\{Q,P\}. \end{aligned}$$

Moreover, Sect. 5.1, below, shows that $\{~,~\}$ is a Lie bracket on ${\mathscr {L}}_{\mathcal {S}}^*$, and so it also satisfies the Jacobi identity,

4.8 $$\begin{aligned} \{P,\{Q,R\}\}+\{Q,\{R,P\}\}+\{R,\{P,Q\}\} \sim 0. \end{aligned}$$

### The BCJ amplitude relations

This section proves the main property of the S bracket, $\{~,~\}$, generalising the *off-shell fundamental BCJ relation* [16]. The on-shell identities follow directly from the off-shell relations, as explained at the end of this section.

#### Proposition

For $P,Q\in {\mathscr {L}}^*$, the *S* bracket $\{~,~\}$ satisfies

4.9 $$\begin{aligned} b(\{P,Q\}) = [b(P),b(Q)], \end{aligned}$$

i.e., *b* maps the *S*-bracket to the Lie bracket.

The proposition is proved in appendix B. It is interesting to observe that the property (4.9) mimics the identity obeyed by the Poisson bracket $\{,\}$ of Hamiltonian vector fields $X_f$: $X_{\{f,g\}} = [X_f,X_g]$ for functions *f* and *g*.

#### Corollary

(Off-shell BCJ relations [16]) Taking $P=i$, a single letter, we obtain4.10where $\alpha (X)=(-1)^{|X|}{\bar{X}}$. Equivalently, we also have

4.11 $$\begin{aligned} \sum _{Q=XY} s_{i,Y} b(XiY) = \left[ i,b(Q)\right] . \end{aligned}$$

#### Proof

(4.10) is immediate. (4.11) follows by showing that

4.12 $$\begin{aligned} \{i,Q\} \sim \sum _{Q=XY} s_{i,Y} XiY. \end{aligned}$$

To prove this identity, use (2.18) to write the RHS (4.12) in the basis of words beginning with the letter *i*,4.13Further manipulations give4.14where we use the property of the shuffle product, (2.8). $\square$

#### Corollary

(BCJ relations) The tree-level partial amplitudes (3.15) satisfy

4.15 $$\begin{aligned} m(\{P,Q\},n) = 0, \end{aligned}$$

where *P* and *Q* are words that partition $1,2,\ldots ,n-1$ into two parts.

#### Proof

By the definition (3.15),

4.16 $$\begin{aligned} m(\{P,Q\},n) =\lim _{s_{PQ}\rightarrow 0} s_{PQ} b(\{P,Q\})= \lim _{s_{PQ}\rightarrow 0}s_{PQ}[b(P),b(Q)] = 0. \end{aligned}$$

The last term vanishes because neither *b*(*P*) nor *b*(*Q*) has a $1/s_{PQ}$ pole. $\square$

The original so called ‘fundamental BCJ relations’ are [35, 36]

4.17 $$\begin{aligned} m(\{i,Q\}n) = \sum _{Q=RS} (k_i\cdot k_S)m(RiSn) = 0, \end{aligned}$$

which follows in this form from (4.11). The first few examples are, [3]

4.18 $$\begin{aligned} \begin{aligned} 0&= m(\{1,23\}4) = s_{12} m(1234) - s_{13} m(1324),\\0&=m(\{1,234\},5) = s_{12} m(12345) - s_{13} m(13245) - s_{13} m(13425) + s_{14} m(14325). \end{aligned} \end{aligned}$$

As observed in [6–15], (4.15) also implies other BCJ relations, such as

4.19 $$\begin{aligned} 0= & {} m(\{12,34\},5) = s_{23} m(1234,5) - s_{13} m(2134,5)\nonumber \\ {}{} & {} - s_{24} m(1243,5) + s_{14} m(2143,5), \end{aligned}$$

while similar formulas using the shuffle product appear in [37–39]. The BCJ relations for Yang–Mills theory were first proven from the field-theory limit of string theory in [37, 38]. By now these relations have been proven for a variety of theories at tree-level. See the recent review [5] and references therein.

## The KLT Matrix as Nested Brackets

This section introduces a canonical KLT map *S*. We find that *S* is the inverse of *b*, and that this implies that $\{~,~\}$ is a Lie bracket. When bases are chosen for ${\mathscr {L}}_n$ and ${\mathscr {L}}_n^*$, the matrix elements of *S* give the ‘generalized KLT matrix’ *S*(*P*|*Q*) proposed in [13]. We prove the conjectured properties of *S*(*P*|*Q*) as well as additional ones.

### The KLT map

Let $\Gamma$ be a Lie monomial, and write it as a nested bracketing. Let $\{\Gamma \} \in {\mathscr {L}}^*$ be obtained by replacing every commutator [ ,  ] in the bracketed expression of $\Gamma$ with a $\{~,~\}$. Since $\{~,~\}$ is antisymmetric, this is well defined. By nested applications of (4.9), it follows from the proposition in the previous section that

#### Proposition

For any Lie monomial $\Gamma$,

5.1 $$\begin{aligned} \Gamma = b(\{\Gamma \}). \end{aligned}$$

These are surprising identities. For example, one can verify that

5.2 $$\begin{aligned} \left[ [1,2],[3,4]\right] = s_{12}s_{34}\bigl (s_{23}b(1234) - s_{24}b(1243) - s_{13} b(2134) + s_{14}b(2143)\bigr ).\nonumber \\ \end{aligned}$$

Define the KLT map $S:{\mathscr {L}}_n \rightarrow {\mathscr {L}}_n^*\otimes {\mathcal {M}}$ by

5.3 $$\begin{aligned} S:\Gamma \mapsto \{\Gamma \}, \end{aligned}$$

for Lie monomials $\Gamma$. It is not obvious that *S* is well defined as a map from ${\mathscr {L}}_n$ to ${\mathscr {L}}_n^*\otimes {\mathcal {M}}$. The map *S* would be well defined provided that $\{~,~\}$ was a Lie bracket. And if *S* is well defined, then *b* is clearly an inverse, since clearly $b(S(\Gamma )) = \Gamma$. This is verified in the following in the proof of the following:

#### Proposition

The maps $b:{\mathscr {L}}^*\rightarrow {\mathscr {L}}$ and $S:{\mathscr {L}}\rightarrow {\mathscr {L}}^*$ are inverses. In particular, *b* is invertible.

#### Proof

Take dual Lyndon bases of ${\mathscr {L}}^*_n$ and ${\mathscr {L}}_n$, as in Sect. 2. Then define a map, $S'$:

5.4 $$\begin{aligned} S': \ell (P) \mapsto \{\ell (P)\}, \end{aligned}$$

for monomials $\ell (P)$ in the given basis of ${\mathscr {L}}_n$. We show that (i) $S'$ and *b* are inverse, and (ii) that the $S'$ in (5.4) is the map, *S*, in (5.3). This proves that (5.3) is well-defined.

(i) By (5.1),
  5.5 $$\begin{aligned} b(S'(\Gamma )) = \sum _{P\in Basis} (\Gamma ,P) b(\{\ell (P)\}) = \sum _{P\in Basis} (\Gamma ,P) \ell (P) = \Gamma . \end{aligned}$$
  Conversely,
  5.6 $$\begin{aligned} S'(b(P)) = \sum _{Q\in Basis} (b(P),Q) \{\ell (Q)\}. \end{aligned}$$
  Expanding $\{\ell (Q)\} \in {\mathscr {L}}^*_n\otimes {\mathcal {M}}$ in the given basis gives (by (2.18)),
  5.7 $$\begin{aligned} \{\ell (Q)\} = \sum _{R\in Basis} (\{\ell (Q)\},\ell (R)) R. \end{aligned}$$
  But notice that
  5.8 $$\begin{aligned} (\{\Gamma \},\Gamma ') = (\{\Gamma \},b(\{\Gamma '\})) =(b(\{\Gamma \}),\{\Gamma '\}) = (\Gamma ,\{\Gamma '\}), \end{aligned}$$
  by the self-adjointness of *b*. Combining (5.7) and (5.8) gives that
  5.9 $$\begin{aligned} S'(b(P)) = \sum _{Q,R \in Basis} b(P,Q) (\ell \{Q\},\ell (R)) R = \sum _{R\in Basis} (P,\ell (R)) R = P. \end{aligned}$$
  So *b* is invertible, with inverse $S'$.
(ii) But $b(\{\Gamma \}) = \Gamma$, by (5.1). So
  5.10 $$\begin{aligned} S'(\Gamma ) = S'(b(\{\Gamma \})) = \{\Gamma \}, \end{aligned}$$
  which shows that $S'$ is the map $S: \Gamma \mapsto \{\Gamma \}$.

$$\square $$

The self-adjointness of *b*, (5.8), implies that the KLT map, *S*, is itself self adjoint:

5.11 $$\begin{aligned} (\Gamma ,S(\Gamma ')) = (S(\Gamma ),\Gamma '). \end{aligned}$$

#### Corollary

$\{~,~\}$ is a Lie bracket on ${\mathscr {L}}^*\otimes {\mathcal {M}}$.

#### Proof

Given that *S* and *b* are inverse, (4.9) shows that $\{~,~\}$ is the pull back of [ ,  ] from ${\mathscr {L}}$ to ${\mathscr {L}}^*$ by $b^{-1}$. So $\{,\}$ is skew and satisfies the Jacobi relation. $\square$

### The generalized and standard KLT matrix

This section finds explicit formulas for the KLT map, *S*, in terms of the matrix elements of *S*. Let us again take dual Lyndon bases: $P\in {\mathscr {L}}_n^*$ for every Lyndon word *P*, and $\ell [P] \in {\mathscr {L}}_n$ for every Lyndon word *P*. Define the *generalized KLT matrix* (introduced in [13]) to be the matrix elements in this basis^5^

5.12 $$\begin{aligned} S^\ell (P|Q):= (S(\ell [P]),\ell [Q]) = (\{\ell [P]\},\ell [Q]). \end{aligned}$$

Some example entries of the generalized KLT matrix are

5.13 $$\begin{aligned} \begin{aligned} S^\ell (12|12)&= s_{12}, S^\ell (12|21) = - s_{12},\\ S^\ell (123|123)&= s_{12}(s_{13}+s_{23}), S^\ell (312|123) = - s_{12}s_{13}. \end{aligned} \end{aligned}$$

It follows from (5.8) that the generalized KLT matrix is symmetric:

5.14 $$\begin{aligned} S^\ell (P|Q) = S^\ell (Q|P). \end{aligned}$$

Moreover, (5.5) implies that the generalized KLT matrix satisfies

5.15 $$\begin{aligned} \ell [P] = \sum _{Q\in Basis} S^\ell (P|Q)\, b(Q). \end{aligned}$$

Or, equivalently,

5.16 $$\begin{aligned} (R,\ell [P]) = \sum _{Q\in Basis} S^\ell (P|Q)\, b(Q|R), \end{aligned}$$

and so, also:

5.17 $$\begin{aligned} b(P|Q) = \sum _{X,Y \in Basis} \, b(P|X)\, S^\ell (X|Y) \, b(Y|Q). \end{aligned}$$

This is what we might call an ‘off shell KLT relation’. We study it further in the next subsection.

The standard KLT matrix arises, as in [13], as a restriction of the generalized one to a fixed basis, and is conventionally written

5.18 $$\begin{aligned} S(P|Q)_i:= S^\ell (iP|iQ), \end{aligned}$$

for some fixed letter *i*, called the ‘fixed leg’. The identity, (5.16), together with the definition (5.18), amount to a purely algebraic proof that the standard KLT matrix is the inverse to the biadjoint scalar BG double current:

5.19 $$\begin{aligned} \sum _R S^\ell (P|R)_i b(iR|iQ) = \delta _{P,Q}. \end{aligned}$$

This is in accord with the discussions of [8, 14]. Moreover, we can easily recover explicit formulas for $S(P|Q)_i$. Using the definition of $\{~,~\}$, and (4.12), we get

5.20 $$\begin{aligned} S^\ell (12\ldots n| 1A)= \prod _{i=2}^n \left( \sum _{C_i=A_iiB_i} s_{i,A_i}\right) , \end{aligned}$$

in agreement with [17–19]. We derive (5.20) in greater detail in appendix C. Finally, the matrix elements can also be efficiently computed using the following recursion relation, originally conjectured in [42].

#### Lemma

The standard KLT matrix can be recursively computed using

5.21 $$\begin{aligned} S(Ai,BiC)_m = k_i\cdot k_{mB}\, S(A,BC)_m, \end{aligned}$$

where *m* is the fixed leg. The recursion is seeded by $S(\emptyset |\emptyset )_m:= 1$.

#### Proof

By definition, $\ell \{Ai\} = \{\ell \{A\},i\}$. Using the formula for $\{P,i\}$, (4.12),

5.22 $$\begin{aligned} \ell \{Ai\} = \sum _{X,Y} s_{i,X}\,(XY,\ell \{A\}) XiY. \end{aligned}$$

Since $S(Ai,BiC)_m = (\ell \{mAi\},\ell (mBiC))$, it follows from (5.22) that

5.23 $$\begin{aligned} S(Ai,BiC)_m = \sum _{X,Y} s_{i,X} (XY,\ell \{mA\}) (XiY,\ell (mBiC)). \end{aligned}$$

The generalized KLT matrix is the matrix of coefficients that arises in the basis expansion of $\ell \{P\}$:

5.24 $$\begin{aligned} \ell \{P\} \sim \sum _{Q\in Basis} (\ell \{P\}, \ell [Q]) Q=\sum _{Q\in Basis} S^\ell (P|Q)Q, \end{aligned}$$

So expanding $\ell \{mA\}$ in a basis,

5.25 $$\begin{aligned} \ell \{mA\} = \sum _P S(A,P)_m\, mP. \end{aligned}$$

Equation (5.23) becomes

5.26 $$\begin{aligned} S(Ai,BiC)_m = \sum _{X,Y,P} s_{i,X} S(A,P)_m (XY,mP) (XiY,\ell (mBiC)). \end{aligned}$$

The only contributions in the sum come from words, $X=mX'$, that begin with the letter *m*. Expanding $\ell (mBiC)$, one sees that $(mX'iY,\ell (mBiC)) = \delta _{X',B}\delta _{Y,C}$. $\square$

### The momentum kernel and the KLT gravity formula

The $(n-2)!$ version of the KLT relation, given by [36, 43], is

5.27 $$\begin{aligned} M_n^\textrm{gravity} = \lim _{s_{1P}\rightarrow 0} \sum _{P,Q} {1\over s_{1P}}{{\mathcal {A}}}(1Pn)S(P|Q)_1 {\tilde{{\mathcal {A}}}}(1Qn), \end{aligned}$$

where the double sum is over permutations of $23\ldots n-1$. In this formula, the gauge theory partial amplitudes $\tilde{{\mathcal {A}}}(1Qn)$ and ${{\mathcal {A}}}(1Pn)$ are understood to have independent sets of polarization vectors, but the same external momenta. It is not immediately obvious that the limit on the RHS of (5.27) is well defined. As we will see, the cancellation of the $1/s_{1P}$ pole on the RHS is best understood using the derivation of $S(P|Q)_1$ in Sect. 5.1, above.

A Lie polynomial version of (5.27) follows immediately from our results in Sect. 5.1. Indeed, (5.17) shows that the off-shell KLT matrix satisfies a KLT-like relation:

5.28 $$\begin{aligned} b(P|Q) = \sum _{R,U} b(P|iR)S(R|U)_i b(iU|Q). \end{aligned}$$

To obtain a relation of the form (5.27), write

5.29 $$\begin{aligned} A(1P,n):= s_{1P} b(1P) = s_{1P}\sum _{R\in S_{n-2}} b(1P|1R)\ell (1R), \end{aligned}$$

for the on-shell ‘Lie polynomial partial amplitude’. We also write

5.30 $$\begin{aligned} M_n = \sum _{P,Q\in S_{n-2}} s_{1P} \,\ell (1P)b(1P|1Q)\ell (1Q) \end{aligned}$$

for the Lie polynomial valued gravity-like amplitude. Notice that, by (3.19), $M_n$ is equivalently written as

5.31 $$\begin{aligned} M_n = s_{12\ldots n-1} \sum _{|\Gamma | = n-1} \frac{\Gamma \otimes \Gamma }{s_\Gamma }. \end{aligned}$$

Then (5.28) implies the KLT relation

5.32 $$\begin{aligned} M_n = \lim _{s_{1P}=0}\sum _{P,Q\in S_{n-2}} {1\over s_{1P}}\, S(P|Q)_1\, A(1Pn)\, \otimes \, A(1Qn). \end{aligned}$$

All the double poles from the right-hand side of (5.27) are manifestly absent in its free Lie algebra version (5.32) due to the generalized KLT matrix property (5.28). The only poles in (5.28) are those that appear in *b*(1*P*|1*Q*).

Another manifestation of the cancellation of the $1/s_{1P}$ pole follows from (5.19), above. This implies that

5.33 $$\begin{aligned} \sum _Q S^{\ell }(1P|1Q)m(1Q,n|R,n) = 0, \end{aligned}$$

where $m(1Q,n|R,n) = \lim _{s_{iP}\rightarrow 0} s_{iP} b(1Q|R)$. The expression vanishes because there is no $1/s_{iP}$ pole in (5.9). This is a remarkable identity, because the components $S(P,Q)_i$ do not have $s_{iP}$ as a factor. On the other hand, *b*(*iP*, *iQ*) certainly *does* have a $1/s_{iP}$ pole. This cancellation is the key reason why the KLT relation, (5.27), is well defined. As discussed below, these results will also make it clear why the existence of a ‘BCJ form’ for gravity (i.e. of the form of $M_n$) is equivalent to existence of the KLT relation of this kind.

To illustrate these surprising cancellations, we end this section with an example of the four-point gravity-like amplitude and its KLT relation. On the one hand, $M_4$ can be written as

5.34 $$\begin{aligned} M_4 ={[[1,2],3]\otimes [[1,2],3] \over s_{12}} + {[[1,3],2]\otimes {[}[1,3],2]\over s_{13}} + {[1,[2,3]]\otimes [1,[2,3]]\over s_{23}}.\nonumber \\ \end{aligned}$$

On the other hand, we can present is as a KLT sum using (5.32) as:

$$\begin{aligned} \begin{aligned} M_4&= \lim _{s_{123}\rightarrow 0} \frac{1}{s_{123}} \big [s_{12}s_{23}A(123,4)\otimes A(123,4) - s_{12}s_{13} A(123,4)\otimes A(132,4) \\&\qquad {}-s_{12}s_{13} A(132,4)\otimes A(123,4) + s_{13}s_{23} A(132,4)\otimes A(132,4)\big ],\\\end{aligned} \end{aligned}$$

where, for example,

5.36 $$\begin{aligned} A(123,4) = \frac{[[1,2],3]}{s_{12}} + \frac{[1,[2,3]]}{s_{23}}. \end{aligned}$$

The poles and numerators in this sum organise to cancel the $s_{123}$ pole and give (5.34) in the limit.

### Discussion: generalized Jacobi identities

The main property of the *S*-bracket $\{~,~\}$ is that $b(\{P,Q\}) = [b(P),b(Q)]$. However, since *b* and *S* are inverse, this also implies the surprising identity,

5.37 $$\begin{aligned} \{P,Q\} = S([b(P),b(Q)]). \end{aligned}$$

The bracket $\{P,Q\}$ is polynomial in the Mandelstam variables, whereas *S*([*b*(*P*), *b*(*Q*)]) is naively a rational function of the Mandelstam variables. As observed in Sect. 5.1, the *S*-bracket is a Lie bracket, and so satisfies

5.38 $$\begin{aligned} \{1,2\}\sim - \{2,1\}\,\qquad \{\{1,2\},3\}+\{\{2,3\},1\}+\{\{3,1\},2\} \sim 0, \end{aligned}$$

as identities in ${\mathscr {L}}^*\otimes {{\mathcal {M}}}$. It follows that $\{~,~\}$ also satisfies

5.39 $$\begin{aligned} \ell \{PiQ\}\sim -\ell \{i\ell [P]Q\} \end{aligned}$$

which can be deduced from two applications of (2.4). This makes it clear that the generalized KLT matrix, $S^\ell (P,Q)$, given in (5.12), satisfies

5.40 $$\begin{aligned} S(XiY|Q) = - S(i\ell [X]Y|Q), \end{aligned}$$

which is called the ‘generalized Jacobi identities’ in [13]. This property of the generalized KLT matrix has no analog for the standard KLT matrix, $S(XiY|Q)_j$, because of the fixed leg *j*.^6^

## The Contact Term Map as a Lie Co-bracket

A series of studies of string theory correlators and BCJ numerators experimentally discovered the so-called *contact term map*, which appears in the action of the BRST operator and in studies of Yang-Mills BCJ numerators [23] in the context of Berends Giele recursion. This section identifies the contact term map as the Lie co-bracket dual to $\{~,~\}$. The main properties of the contact map follow directly.

### Definition

(*Contact term map*). The contact term map, *C*, is the dual of $\{~,~\}$. For a Lie monomial, $\Gamma$,

6.1 $$\begin{aligned} (P\otimes Q, C(\Gamma )):= (\{P,Q\}, \Gamma ). \end{aligned}$$

An explicit formula for $C(\Gamma )$ follows from (6.1) by choosing a basis, for example:

6.2 $$\begin{aligned} C(\Gamma ) = \sum _{P,Q} (\{P,Q\},\Gamma )\; \ell [P]\otimes \ell [Q], \end{aligned}$$

where the sum is over a Lyndon basis of ${\mathscr {L}}^*$.

It is convenient to write

6.3 $$\begin{aligned} P\wedge Q:= P\otimes Q - Q\otimes P. \end{aligned}$$

Then the first few examples of the map *C* are

6.4 $$\begin{aligned} \begin{aligned} ~&C([1,2]) = (k_1\cdot k_2) (1\wedge 2 ),\\~&C([1,[2,3]]) = (k_2\cdot k_3)\left( [1,2]\wedge 3 + 2\wedge [1,3]\right) + (k_{1}\cdot k_{23}) \left( 1 \wedge [2,3]\right) . \end{aligned} \end{aligned}$$

*C* satisfies the *dual Jacobi identity*,

6.5 $$\begin{aligned} (C\otimes Id)\circ C - (Id\otimes C)\circ C - (Id\otimes A) \circ (C\otimes 1) \circ C = 0, \end{aligned}$$

where *A* is the swap map: $X\otimes Y\mapsto Y\otimes X$. This dual Jacobi identity follows immediately from the Jacobi identity satisfied by the *S*-bracket. Moreover, recall that

6.6 $$\begin{aligned} b(\{P,Q\}) = [b(P),b(Q)]. \end{aligned}$$

This can be used to show:

### Lemma

*C* satisfies [23]

6.7 $$\begin{aligned} C(b(P)) = \sum _{P=XY} b(X)\wedge b(Y). \end{aligned}$$

### Proof

(6.6) implies that

6.8 $$\begin{aligned} (P\otimes Q, C(b(R))) = ([b(P),b(Q)], R). \end{aligned}$$

The RHS can be expanded by deconcatenation (as in the derivation of (3.10)):

6.9 $$\begin{aligned} RHS = \sum _{R=XY} (b(P),X)(b(Q),Y) - (X\leftrightarrow Y). \end{aligned}$$

But *b* is self-adjoint, and so

6.10 $$\begin{aligned} (P\otimes Q,C(b(R))) = \sum _{R=XY} (P,b(X))(Q,b(Y)) - (X\leftrightarrow Y), \end{aligned}$$

and this is equivalent to (6.7). (6.7) can also be checked using the formula, (6.2). $\square$

We finish this section by deriving a recursive formula for *C*. First define the standard extension of the adjoint representation of ${\mathscr {L}}$ to ${\mathscr {L}}\otimes {\mathscr {L}}$:

$$\begin{aligned} \begin{aligned} \left[ P,X\otimes Y\right]&:= \left[ P,X\right] \otimes Y + X\otimes [P,Y],\\\left[ X\otimes Y,Q\right]&:= [X,Q]\otimes Y + X\otimes [Y,Q]. \end{aligned} \end{aligned}$$

This makes ${\mathscr {L}}\otimes {\mathscr {L}}$ into an adjoint representation of ${\mathscr {L}}$.

### Lemma

(Recursion). For $\Gamma _1,\Gamma _2\in {\mathscr {L}}$, the action of *C* on $\left[ \Gamma _1,\Gamma _2\right]$ is given by

6.11 $$\begin{aligned} C([\Gamma _1,\Gamma _2]):= k_{1}\cdot k_{2}\, \Gamma _1\wedge \Gamma _2+[C(\Gamma _1), \Gamma _2] + \left[ \Gamma _1,C(\Gamma _2)\right] , \end{aligned}$$

where $k_1$ and $k_2$ are the momenta associated to $\Gamma _1$ and $\Gamma _2$. With $C(i):=0$, (6.11) can be taken as a definition of *C*, as in [23].

### Proof

This is a consequence of the identity, (B.5). We again use deconcatenation, as in (6.8), to write

6.12 $$\begin{aligned} (\{P,Q\},[\Gamma _1,\Gamma _2]) = (\delta \{P,Q\}, \Gamma _1\otimes \Gamma _2) - (1\leftrightarrow 2). \end{aligned}$$

Then (B.5) gives

6.13 $$\begin{aligned} \begin{aligned} (P\otimes Q,C(\left[ \Gamma _1,\Gamma _2\right] ))&= k_{P}\cdot k_{Q} (P,\Gamma _1)(Q,\Gamma _2) \\&+ \sum _{P=XY} (X,\Gamma _1)(\{Y,Q\},\Gamma _2)\\ {}&- \sum _{Q=XY} (\{P,X\},\Gamma _1)(Y,\Gamma _2) - (1\leftrightarrow 2). \end{aligned} \end{aligned}$$

But $(\{Y,Q\},\Gamma _2) = (Y\otimes Q,C(\Gamma _2))$ and $(\{P,X\},\Gamma _1) = (P\otimes X, C(\Gamma _1))$, and so (6.13) is equivalent to (6.11). $\square$

The recursive relation, (6.11), can be solved to find an explicit formula for $C(\Gamma )$. To see this, write

6.14 $$\begin{aligned} D(\Gamma ):= k_1\cdot k_2\, \Gamma _1\wedge \Gamma _2, \end{aligned}$$

for Lie monomials $\Gamma = \left[ \Gamma _1,\Gamma _2\right]$. Nesting (6.11) then leads to a sum over the edges in the tree $T_\Gamma$. For $I \in \Gamma$ an edge in the tree $T_\Gamma$, let $\Gamma _I$ be the associated Lie monomial. For example, if $\Gamma =[[1,2],[3,4]]$, then $\Gamma _{12} = [1,2]$. Then the solution to the recursion, (6.11), is

6.15 $$\begin{aligned} C(\Gamma ) = \sum _{I\in \Gamma } \Gamma /\Gamma _{I}[D(\Gamma _I)], \end{aligned}$$

where $\Gamma /\Gamma _{I}[D(\Gamma _I)]$ denotes the replacement, in $\Gamma$, of $\Gamma _I$ by $D(\Gamma _I)$. For example, if $\Gamma =[[1,2],[3,4]]$, then $C(\Gamma )$ is

6.16 $$\begin{aligned} C(\Gamma ) = D(\Gamma ) + \Gamma /\Gamma _{12}[D(\Gamma _{12})] + \Gamma /\Gamma _{34}[D(\Gamma _{34})], \end{aligned}$$

where

6.17 $$\begin{aligned} \Gamma /\Gamma _{34}[D(\Gamma _{34})] = s_{34} [[1,2],3\otimes 4] = s_{34} \left( [[1,2],3]\otimes 4 + 3\otimes [[1,2],4] \right) , \end{aligned}$$

and

6.18 $$\begin{aligned} D(\Gamma ) = k_{12}\cdot k_{34} \, [1,2]\wedge [3,4]. \end{aligned}$$

We will see applications of these structures in the discussion of numerators in Sect. 7.5.

## BCJ Numerators

In the previous sections we used Berends–Giele recursion and the properties of Lie polynomials to relations for our Lie polynomial version of biadjoint scalar theory. When dressed with BCJ numerators these results apply to other gauge theories. In this section, we first argue that any gauge theory that admits the Berends–Giele framework can be understood in this way. There are clear distinctions between the behaviour of numerators for on-shell amplitudes, versus those for the partially off-shell Berends–Giele multiparticle fields; for a given Berends–Giele description we will see that numerators are unique, but ambiguities arise due to gauge transformations and field redefinitions. We then use this to study examples of numerators $N^\textrm{theory}_\Gamma$ for different theories.

### BCJ numerators off-shell

Many gauge theories have perturbation expansions that can be expanded in colour factors of the form (2.13); this is the case for any gauge theory whose Lagrangian is second order and single trace in the Lie algebra. The tree amplitudes for such gauge theory can be solved using Berends–Giele recursion for ${\mathscr {L}}$-valued fields. The recursion produces colour-ordered Berends–Giele currents, $B^\textrm{theory}(P)$, that give the partial amplitudes of the theory as

7.1 $$\begin{aligned} \mathcal {A}^\textrm{theory}(Pn)=\lim _{s_P\rightarrow 0}s_P B^\textrm{theory}(P)\cdot \epsilon _n, \end{aligned}$$

where $\epsilon _n$ is the polarization of the *n*th particle. These partial amplitudes satisfy shuffle relations as a consequence of the *B*(*P*) being defined as functions of $P\in {\mathscr {L}}^*$. The amplitudes $\mathcal {A}(Pn)$ obtained this way are necessarily invariant under both field redefinitions and gauge transformations. However, the Berends–Giele currents, $B^\textrm{theory}(P)$, are not invariant. Different choices of gauge fixing and field redefinition lead to different Berends–Giele recursions and different associated currents.

We now study the properties of these $B^\textrm{theory}(P)$ for a general gauge theory, and in the next section derive necessary conditions for this theory to have KLT relations. Dropping the superscript ‘theory’, fix some gauge theory, and let *B*(*P*) be the BG currents of the theory obtained using some choices of gauge fixing and field redefinitions. We derive *B*(*P*) using the ${\mathscr {L}}$-valued recursion method given for biadjoint scalar in Sect. 3 and for Yang-Mills in appendix A. This means that the *B*(*P*) are functions of ${\mathscr {L}}^*$ and so satisfy7.2for $R,S\ne \emptyset$. This allows us to define

7.3 $$\begin{aligned} {\tilde{N}}_\Gamma := B(\{\Gamma \})\,, \end{aligned}$$

where we recall that $\{\Gamma \}$ is only well-defined as an element of ${\mathscr {L}}^*$. We call the ${\tilde{N}}_\Gamma$ ‘off-shell BCJ numerators’ for the currents *B*(*P*). The map, ${\tilde{N}}: {\mathscr {L}}\rightarrow {\mathcal {K}}$, given by

7.4 $$\begin{aligned} {\tilde{N}}: \Gamma \mapsto {\tilde{N}}_\Gamma \end{aligned}$$

is a homomorphism, since the *S*-bracket is a Lie bracket. Moreover, the ${\tilde{N}}_\Gamma$ defined by (7.3) are unique. Note that the numerators ${\tilde{N}}_\Gamma$ may have free indices that we do not write (e.g. for YM, ${\tilde{N}}_\Gamma$ has a free gluon polarization index). It follows from the results of Sect. 5 that ${\tilde{N}}$ relates *B*(*P*) to the biadjoint scalar *b*(*P*) by

7.5 $$\begin{aligned} B(P)={\tilde{N}}(b(P)). \end{aligned}$$

To be more explicit, we write (7.5) and (7.3) in a basis. Using (5.24), the ${\tilde{N}}_\Gamma$ for a basis of ${\mathscr {L}}_n$ is given by

7.6 $$\begin{aligned} {\tilde{N}}_{\ell [P]}= B(\ell \{P\})=\sum _{Q\in Basis}S^\ell (P|Q) B(Q)\,. \end{aligned}$$

If we multiply this by $s_P$, contract free indices with the *n*th particle polarization, and take the limit $s_P\rightarrow 0$, (7.6) becomes the formula for on-shell amplitude BCJ numerators given in [44] (see also [42]). Also, in this basis, the relation (7.5) reads

7.7 $$\begin{aligned} \sum _{Q\in Basis} {\tilde{N}}_{\ell [Q]} \, b(P|Q)= \sum _{Q,R\in Basis} S^\ell (Q|R) \, B(R)\, b(P|Q)\,. \end{aligned}$$

The off-shell BCJ numerators defined by ${\tilde{N}}$ in turn define ‘on-shell BCJ numerators’ for the amplitude, given by

7.8 $$\begin{aligned} N_\Gamma = \lim _{s_P\rightarrow 0} {\tilde{N}}_\Gamma \cdot \epsilon _n. \end{aligned}$$

This again defines a homomorphism $N: \Gamma \mapsto N_\Gamma$ out of ${\mathscr {L}}_n$. Moreover, by (7.1) and (7.5), the partial amplitudes of the theory are given by

7.9 $$\begin{aligned} {{\mathcal {A}}}(Pn) = N(m(Pn)), \end{aligned}$$

where *m*(*Pn*) are the ${\mathscr {L}}$-valued biadjoint scalar amplitudes ($m(Pn) = s_P b(P)$). Unlike the ${\tilde{N}}_\Gamma$, the numerators $N_\Gamma$ are subject to a new gauge freedom, given by replacements

7.10 $$\begin{aligned} N_\Gamma \mapsto N_\Gamma + N^\textrm{gauge}_\Gamma , \end{aligned}$$

where the gauge freedom is spanned by ‘trivial’ numerators of the form

7.11 $$\begin{aligned} N^\textrm{gauge}_\Gamma = \sum _{R,S} C_{R,S} (\{R,S\},\Gamma ), \end{aligned}$$

for some arbitrary kinematic functions $C_{R,S} \in \mathcal {K}$. These trivial numerators give

7.12 $$\begin{aligned} N_\Gamma ^\textrm{gauge} ( b(P))= \sum _{R,S} C_{R,S} b(P|\{R,S\})= \sum _{R,S} C_{R,S} (P, [b(R),b(S)])\,. \end{aligned}$$

This contribution vanishes on-shell, because the RHS (7.12) has no $1/s_P$ pole, and so vanishes when multiplied by $s_P$, in the $s_P\rightarrow 0$ limit. This gauge freedom, in a different guise, led to the original discovery of the BCJ relations in [3], where it was argued that there are $(n-2)!-(n-3)!$ independent pure gauge numerators of this form. However, (7.12) shows that these no longer vanish off-shell. The off-shell numerators are therefore not subject to the freedom, (7.11), and are unique once a choice of Berends Giele formulation has been made.

### BCJ numerators and KLT

Two gauge theories with BG currents *B*(*P*) and $B'(P)$ satisfy an off-shell KLT relation if we can write

7.13 $$\begin{aligned} M=\sum _{P,Q\in Basis} S^\ell (P|Q)B(P) B'(Q)\,, \end{aligned}$$

for *M* the current for some gravity-like theory. If *M* arises from a local field theory, the current *M* must have the appropriate kinematic poles for factorization. This places constraints on the RHS of (7.13), which must have the same poles. Note that

7.14 $$\begin{aligned} \sum _P S^\ell (P|Q) B(P) = {\tilde{N}} (\ell [Q]). \end{aligned}$$

So the only poles on the RHS of (7.13) come from $B'(Q)=N'(b(Q))$ and ${\tilde{N}}$. To guarantee the correct poles, we demand that the off-shell numerators ${\tilde{N}}$ and ${\tilde{N}}'$ have no kinematic poles. We will say that ${\tilde{N}}$ is local if it has no poles in the Mandelstam variables.

For a generic choice of gauge theory and currents *B*(*P*), there is no reason to expect that the *off-shell* numerators obtained from (7.3) will be local. For example, an obstruction to locality for Yang-Mills Berends Giele currents in Lorenz gauge is identified in [15, 23, 28].^7^ However, if the off-shell numerators ${\tilde{N}}$ are local, they restrict to give local on-shell numerators for the amplitudes of the theory, using (7.8).^8^ Local BCJ numerators are known for both the nonlinear sigma model (NLSM), [42–44] and for (super-)Yang-Mills [11, 15, 23, 44–47]. See [5] for a review.

If the off-shell numerators, ${\tilde{N}}$, of a gauge theory are local, this implies that the theory satisfies the on-shell BCJ relations. Indeed,

7.15 $$\begin{aligned} B(\{P,Q\})={\tilde{N}}(b(\{P,Q\})). \end{aligned}$$

If ${\tilde{N}}$ is local, $B(\{P,Q\})$ will have no $1/s_{PQ}$ pole, since $b(\{P,Q\})$ has no $1/s_{PQ}$ pole. It follows that the partial amplitudes associated to *B*(*P*) satisfy

7.16 $$\begin{aligned} A^\textrm{theory}(\{P,Q\},n)= \lim _{s_{PQ}\rightarrow 0} s_{PQ} B^\textrm{theory} (\{P,Q\})\cdot \epsilon _n=0, \end{aligned}$$

which are the fundamental BCJ relations. Given this, we propose:

**Conjecture:** If a theory’s partial amplitudes satisfy the BCJ relations, then there exists a field redefinition and gauge fixing of its Berends–Giele recursion so that it has *local* off-shell numerators.

The following subsections review what is known in the cases of the biadjoint scalar, NLSM, Yang-Mills, and Z theory.

### Biadjoint scalar

The Berends–Giele recursion for the biadjoint scalar theory was studied in Sect. 3, where we obtained ‘currents’,

7.17 $$\begin{aligned} b(P|Q) = (b(P),Q), \end{aligned}$$

where recall that *b*(*P*) takes values in ${\mathscr {L}}$, and ( ,  ) is the pairing between ${\mathscr {L}}$ and ${\mathscr {L}}^*$. This can be written as

7.18 $$\begin{aligned} b(P|Q) = N(Q) (b(P)), \end{aligned}$$

where *N*(*Q*) is the homomorphism $\Gamma \mapsto (Q,\Gamma )$. In other words, the off-shell numerators for these currents is

7.19 $$\begin{aligned} {\tilde{N}}_\Gamma (Q) = (Q,\Gamma ). \end{aligned}$$

The on-shell numerators are then

7.20 $$\begin{aligned} N_\Gamma (Q) = (Q,\Gamma ). \end{aligned}$$

With these numerators, the biadjoint amplitudes *m*(*Pn*|*Qn*) are then given by,

7.21 $$\begin{aligned} m(Pn|Qn) = \lim _{s_P\rightarrow 0}s_P(P, b(Q))) = \lim _{s_P\rightarrow 0} s_P \sum _\Gamma \frac{(P,\Gamma ) N_\Gamma (Q)}{s_\Gamma }, \end{aligned}$$

As observed in Sect. 4.2, the Kleiss-Kuijf relations follow from Ree’s theorem as  because *b*(*Q*) is a Lie polynomial, while the BCJ relations follow from the $\{,\}$-bracket.

Examples of biadjoint amplitudes with the numerators *N*(*Q*) are obtained from *b*(123) and *b*(1234):

7.22 $$\begin{aligned} \begin{aligned} m(1234|1234)&= {\langle 123,[[1,2],3]\rangle \over s_{12}} + {\langle 123,[1,[2,3]]\rangle \over s_{23}} ={1\over s_{12}}+{1\over s_{23}}, \\m(12345|14235)&= {\langle 1234, [ [ [ 1, 4 ], 2 ], 3 ] \rangle \over s_{14} s_{124}} + {\langle 1234, [ [ 1, [ 4, 2 ] ], 3 ] \rangle \over s_{124} s_{24}}\\&+ {\langle 1234, [ [ 1, 4 ], [ 2, 3 ] ] \rangle \over s_{14} s_{23}}\\&+ {\langle 1234, [ 1, [ [ 4, 2 ], 3 ] ] \rangle \over s_{24} s_{234}} + {\langle 1234, [ 1, [ 4, [ 2, 3 ] ] ] \rangle \over s_{234} s_{23}}= - {1\over s_{23}s_{234}}, \end{aligned} \end{aligned}$$

where the expansion of *b*(123) and *b*(1234) can be found in (3.18).

**Remark.** This perspective on biadjoint scalar theory was developed in a series of papers. [14] showed that these amplitudes could be derived from solving the biadjoint scalar field equations to get Berends–Giele ‘currents’ *b*(*P*|*Q*). In [13], these ‘currents’ were rewritten in terms of *b*(*P*), with values in planar binary trees. Following [8], it was also pointed out in [14] that this Berends–Giele multiparticle field *b*(*iP*|*iQ*) gives rise to an efficient algorithm to compute the inverse of the KLT matrix $S(P|Q)_i$, but no proof was given for this statement. The statement that the KLT matrix is the inverse to the “biadjoint amplitudes” had already been argued on general grounds in [20–22]. The direct proofs of this statement and of the recursion for $S(P|Q)_i$ conjectured in [14], are given above in Sect. 5.

### NLSM

NLSM amplitudes [42] can be studied by BG recursion, as above for biadjoint scalar theory. Several authors have suggested the following formula the (off-shell) BCJ numerators for NLSM:

7.23 $$\begin{aligned} {\tilde{N}}^\textrm{NLSM}_\Gamma := \sum _P (P,\Gamma )S^\ell (P|P), \quad \hbox {for } {|\Gamma |}=n \end{aligned}$$

where the sum is over a basis. It is clear that ${\tilde{N}}^\textrm{NLSM}$ is a homomorphism out of ${\mathscr {L}}_n$. We refer to [26] for a proof of (7.23) using methods adapted to $\mathcal{M}_{0,n}$; alternatively, this result also follows from the Berends–Giele currents produced by the Lagrangian for NLSM introduced in [48]. The NLSM amplitudes are then given by

7.24 $$\begin{aligned} A^\textrm{NLSM}(Pn) = \lim _{s_P\rightarrow 0}s_P {\tilde{N}}^\textrm{NLSM}\big (b(P)\big ). \end{aligned}$$

The numerators at $n=4$ points are given by

7.25 $$\begin{aligned} \begin{aligned} N^\textrm{NLSM}_{[[1,2],3]}&= s_{12}(s_{23}+s_{13}),\\N^\textrm{NLSM}_{[1,[2,3]]}&= s_{12}(s_{23}+s_{13}) - s_{13}(s_{23}+s_{12}) = s_{23}(s_{12}-s_{13}). \end{aligned} \end{aligned}$$

Substituting this into (7.24) then gives the 4-point amplitude:

7.26 $$\begin{aligned} A^\textrm{NLSM}(1234) = s_{123}{{\tilde{N}}}^\textrm{NLSM}( b(123)) = s_{12}+s_{23}. \end{aligned}$$

From the existence of the map ${\tilde{N}}^\textrm{NLSM}$, and the results in Sect. 4, it follows that the KK and BCJ relations are automatically satisfied by the NLSM amplitudes, as was first proved using amplitudes methods in [49]. In [42], master BCJ numerators with fixed legs 1 and *n* of the NLSM amplitudes were conjectured to be $N_{1|P|n}=(-1)^{n/2}S(P|P)_1$ for even *n*. This follows from (7.23).

### Super-Yang–Mills

String theory OPEs (or supersymmetric BG recursion) can be used to recursively compute local SYM multiparticle superfields $\{A^\Gamma _\alpha ,A^\mu _\Gamma ,W^\alpha _\Gamma ,F^{\mu \nu }_\Gamma \}$, $\mu ,\nu =1,\ldots ,10$ in the BCJ gauge which are labelled by Lie monomials $\Gamma \in {\mathscr {L}}$ [6, 23, 28]. As demonstrated in [23, 28], the words labelling these superfields satisfy ‘generalized Jacobi identities’ (as in Sect. 5.4). For example,

7.27 $$\begin{aligned} A^\mu _{[[1,2],[3,4]]} = A^\mu _{[[[1,2],3],4]} -A^\mu _{[[[1,2],4],3]}. \end{aligned}$$

This leads to a proposal for local off-shell BCJ numerators $\tilde{N}_\mu ^\textrm{SYM}$ from which SYM tree amplitudes arise from

7.28 $$\begin{aligned} \mathcal {A}^\textrm{SYM}(Pn) =A^\mu _n\lim _{s_P\rightarrow 0} s_P {\tilde{N}}_\mu ^\textrm{SYM} \big ( b(P)\big ), \end{aligned}$$

where $A^\mu _n$ is the polarization vector of the *n*th particle while the action of ${{\tilde{N}}}_\mu ^\textrm{SYM}$ on the Lie polynomials $\Gamma$ in (3.10) is given by

7.29 $$\begin{aligned} A^\mu _n{{\tilde{N}}}_{\mu \Gamma }^\textrm{SYM}:= A_{n\mu } A^\mu _\Gamma \,, \end{aligned}$$

in terms of the $\theta =0$ component of the superfield $A^m_\Gamma$. This representation manifestly satisfies the BCJ identities. For example, the five-point color-ordered amplitudes in the Kleiss–Kuijf basis following from the maps (7.28) and (7.29) are given by

7.30 $$\begin{aligned} \begin{aligned} \mathcal {A}(12345) = \Big ({A^\mu _{[ [ [ 1, 2 ], 3 ], 4 ]} \over s_{12} s_{45}} + {A^\mu _{[ 1, [ [ 2, 3 ], 4 ] ]} \over s_{23} s_{51}} + {A^\mu _{[ [ 1, 2 ], [ 3, 4 ] ]} \over s_{12} s_{34}} + {A^\mu _{[ [ 1, [ 2, 3 ] ], 4 ]} \over s_{45} s_{23}} + {A^\mu _{[ 1, [ 2, [ 3, 4 ] ] ]} \over s_{51} s_{34}}\Big ) A_{5\mu } \end{aligned}\nonumber \\ \end{aligned}$$

together with the other 5 permutations of 2, 3, 4. The BCJ numerator identities are manifestly satisfied. For example, comparing the above parametrization with the one in [3] leads to

7.31 $$\begin{aligned} n_3 = A^m_{[ [ 1, 2 ], [ 3, 4 ] ]}A^m_5,\qquad n_5 = A^m_{[ 1, [ 2, [ 3, 4 ] ] ]}A^m_5,\qquad n_8 = A^m_{[ [ 1, [ 4, 3 ] ], 2 ]}A^m_5,\qquad \end{aligned}$$

from which the identity $n_3-n_5+n_8=0$ can easily be verified.

In section 6.1.2 of [23] two alternatives were presented for the map ${{\tilde{N}}}_\mu ^\textrm{SYM}$ writing for $\Gamma =[\Gamma _1,\Gamma _2]$

7.32 $$\begin{aligned} A^\mu _n{{\tilde{N}}}_{\mu \Gamma }^\textrm{SYM}:= \langle V_{\Gamma _1}V_{\Gamma _2}V_n\rangle = H'_{\Gamma _1,\Gamma _2,n} \end{aligned}$$

where the superfields $V_\Gamma$ are defined in [6, 23] and are related to the unintegrated vertex operator in the pure spinor formalism and $\langle \cdot \rangle$ represents the pure spinor bracket from [24]. The superfields $H'_{P,Q,R}$ are computed to all orders in [23].

The contact term map *C* plays a key role in the definition of the $V_\Gamma$ [6, 23, 28]. Associated to the $V_\Gamma$ are SYM Berends–Giele currents, $M_P = V(b(P))$, where $V(\Gamma ):= V_\Gamma$. For example,

7.33 $$\begin{aligned} M_1 = V_1, \quad M_{12} = {V_{[1,2]}\over s_{12}},\quad M_{123} = {V_{[[1,2],3]}\over s_{12}s_{123}} + {V_{[1,[2,3]]}\over s_{23}s_{123}}. \end{aligned}$$

The equation of motion of $M_{P}$ under the action of the pure spinor BRST charge *Q* is computed in examples and is conjecturally

7.34 $$\begin{aligned} QM_P = \sum _{XY=P}M_XM_Y. \end{aligned}$$

Whereas the equation of motion for $V_\Gamma$ is conjecturally

7.35 $$\begin{aligned} QV_\Gamma = (V\otimes V, C(\Gamma )) = \sum _{P,Q} V_{\ell (P)}V_{\ell (Q)} (P\otimes Q,C(\Gamma )), \end{aligned}$$

where the sum is over a basis. For example,

7.36 $$\begin{aligned} \begin{aligned} QV_{[1,2]}&=(k_1\cdot k_2) V_1V_2,\\QV_{[[1,2],3]}&= (k_1\cdot k_2)(V_{[1,3]}V_2 + V_1V_{[2,3]}) + (k_{12}\cdot k_3)V_{[1,2]}V_3,\\QV_{[1,[2,3]]}&= (k_2\cdot k_3)\big (V_{[1,2]}V_3 + V_2V_{[1,3]}\big ) + (k_1\cdot k_{23})V_1V_{[2,3]}, \end{aligned} \end{aligned}$$

Equation (6.7) above, allows one to prove (7.34) as an immediate consequence of equation (7.35), as explained in [23]. This was previously only known to be true examples. It remains to prove (7.35) itself, using string theory methods.

### Z-theory and the open superstring

In order to upgrade the discussion in the previous subsection to the open superstring with ${\alpha ^{\prime }}$ corrections we will exploit non-abelian Z-theory to evaluate ${\alpha ^{\prime }}$ expansions of open string disk integrals. Z-theory disk integrals can be computed via the Berends–Giele method as

7.37 $$\begin{aligned} Z(P,n|Q,n) = \lim _{s_P\rightarrow 0}s_P b^{\alpha ^{\prime }}(P|Q) \end{aligned}$$

where the Berends–Giele currents $b^{\alpha ^{\prime }}(P|Q)$ are computed using the equations of motion of the non-abelian Z-theory [50]. In principle, these can be obtained from ‘currents’ $b^{\alpha ^{\prime }}(P)$ with values in ${\mathscr {L}}_n$ by computing ${\alpha ^{\prime }}$ corrections to the Catalan expansion (3.10) and defining

7.38 $$\begin{aligned} b^{\alpha ^{\prime }}(P|Q) = (b^{\alpha ^{\prime }}(P), Q) \end{aligned}$$

which together with (7.37) implies that Z-theory admits a free Lie algebra representation. The $b^{\alpha ^{\prime }}(P|Q)$ are known up to ${\alpha ^{\prime }}^7$ order following [30]. Using this result, we can compute $b^{\alpha ^{\prime }}(P)$ to the same order. The first few orders are given by

7.39 $$\begin{aligned} \begin{aligned} s_P b^{\alpha ^{\prime }}(P)&= \sum _{XY=P}[b^{\alpha ^{\prime }}(X),b^{\alpha ^{\prime }}(Y)] \\&\quad + {\alpha ^{\prime }}^2\zeta _2\sum _{XYZ=P}k_X\cdot k_Y[b^{\alpha ^{\prime }}(X),[b^{\alpha ^{\prime }}(Z),b^{\alpha ^{\prime }}(Y)]]\\&\quad - {\alpha ^{\prime }}^2\zeta _2\sum _{XYZ=P}k_Y\cdot k_Z[[b^{\alpha ^{\prime }}(X),b^{\alpha ^{\prime }}(Y)],b^{\alpha ^{\prime }}(Z)]\\&\quad + {\alpha ^{\prime }}^2\zeta _2\sum _{XYZW=P}[[b^{\alpha ^{\prime }}(X),b^{\alpha ^{\prime }}(Y)],[b^{\alpha ^{\prime }}(W),b^{\alpha ^{\prime }}(Z)]]\\&\quad - {\alpha ^{\prime }}^2\zeta _2\sum _{XYZW=P}[[b^{\alpha ^{\prime }}(X),b^{\alpha ^{\prime }}(Z)],[b^{\alpha ^{\prime }}(W),b^{\alpha ^{\prime }}(Y)]] +{{\mathcal {O}}}({\alpha ^{\prime }}^3) \end{aligned} \end{aligned}$$

and so on. It remains to discover a simple way to extend this calculation to all orders in ${\alpha ^{\prime }}$.

Note that $b^{\alpha ^{\prime }}(P|Q) \ne b^{\alpha ^{\prime }}(Q|P)$. Indeed, $b^{\alpha ^{\prime }}(P)$ is not a function of $P\in {\mathscr {L}}_n^*$, but rather satisfies shuffle-like relations that are twisted by the monodromies of the Z-theory disk integrals, as explained in [50]. Whereas we take the second factor in $b^{\alpha ^{\prime }}(P|Q)$ to belong to $Q\in {\mathscr {L}}_n^*$. It follows that , because $b^{\alpha ^{\prime }}(P)$ is valued in ${\mathscr {L}}_n$.

Finally, we conjecture that the full open superstring disk amplitudes including ${\alpha ^{\prime }}$ corrections are given by using the super-Yang-Mills numerators ${{\tilde{N}}}^\textrm{SYM}$ from the previous subsection:

$$\begin{aligned} \begin{aligned} \mathcal {A}^\textrm{string}(P,n)&= A_n^\mu \lim _{s_P\rightarrow 0} s_P {\tilde{N}}_\mu ^\textrm{SYM}( b^{\alpha ^{\prime }}(P) ). \end{aligned} \end{aligned}$$

This proposal has been verified for the bosonic components of the amplitudes at low *n*.

## Conclusions

We have seen that many nontrivial properties of gauge theory tree amplitudes follow from the properties of ${\mathscr {L}}$ and its dual. Moreover, these results are particularly important for the study of gauge theories that have KLT relations. This lead us to our main conjecture:

**Conjecture:** If a theory’s partial amplitudes satisfy the BCJ relations, then there exists a field redefinition and gauge fixing of its Berends–Giele recursion so that it has *local* off-shell numerators.

If a theory has local off-shell BCJ numerators, we showed above that this immediately implies that it satisfies the KLT relations to give the amplitudes of some gravity-like theory. We showed that, for a given gauge theory, every formulation of Berends–Giele recursion of that theory produce a unique set of off-shell numerators ${\tilde{N}}$. It is a well-posed question to ask whether there exists a field redefinition and gauge fixing of BG recursion such that the $\tilde{N}$ are local, and we hope that this question can be answered systematically.

We conclude by explaining how our results bear on three outstanding problems in this area: the existence of so-called ‘kinematic algebras’, the existence of BCJ numerators for string theory, and the extension of KLT relations to all orders in perturbation theory.

**Kinematic algebras.** An ongoing topic of research is the identification of the kinematic algebra for a given theory, see the review [5]. Our results in 7 suggests a possible route to defining the kinematic algebra systematically. For a given gauge theory, BG recursion leads to some currents, *B*(*P*), and their associated off-shell numerators ${\tilde{N}}_\Gamma$. These ${\tilde{N}}_\Gamma$ satisfy

8.1 $$\begin{aligned} {\tilde{N}}_{[\Gamma _1,\Gamma _2]} = B(\{[\Gamma _1,\Gamma _2]\}). \end{aligned}$$

The ${\tilde{N}}_\Gamma$ take values in some (off-shell) kinematic space $\mathcal {K}$. Motivated by (8.1), we can define a bracket

8.2 $$\begin{aligned} \{{\tilde{N}}_{\Gamma _1},{\tilde{N}}_{\Gamma _2}\}_\mathcal {K}:= B(\{[\Gamma _1,\Gamma _2]\}), \end{aligned}$$

where $\{~,~\}_\mathcal {K}$ is defined the image of ${\tilde{N}}$ in $\mathcal {K}$. This $\{~,~\}_\mathcal {K}$ is a Lie bracket because $\{~,~\}$ is Lie and ${\tilde{N}}$ is a homomorphism by construction. Imposing that $\{~,~\}_\mathcal {K}$ is linear in Mandelstam variables, this definition implies a BCJ-like relation,

8.3 $$\begin{aligned} B(\{P,Q\}) = \{B(P),B(Q)\}_\mathcal {K}, \end{aligned}$$

where, by linearity,

8.4 $$\begin{aligned} \{B(P),B(Q)\}_\mathcal {K}:= \sum \frac{(P,\Gamma _1)}{s_{\Gamma _1}} \frac{(Q,\Gamma _2)}{s_{\Gamma _2}} \{{\tilde{N}}_{\Gamma _1},\tilde{N}_{\Gamma _2}\}_\mathcal {K}. \end{aligned}$$

If the off-shell numerators ${\tilde{N}}$ are *local*, then The definition (8.2), together with (8.1), implies that the numerators in a basis are given by complete bracketings of $\{~,~\}_\mathcal {K}$:

8.5 $$\begin{aligned} {\tilde{N}}_{\Gamma } = \{\Gamma \}_\mathcal {K}. \end{aligned}$$

Moreover, if the ${\tilde{N}}$ are *local*, then the numerators of the amplitude are given by

8.6 $$\begin{aligned} N_{\Gamma } = \lim _{s_P\rightarrow 0} \{\Gamma \}_\mathcal {K}\cdot \epsilon _n. \end{aligned}$$

In this case, it would therefore be reasonable to call $\{~,~\}_\mathcal {K}$ the “kinematic algebra” of the theory. This suggests that, for a theory satisfying BCJ relations, the existence of a kinematic algebra is another consequence of our main conjecture proposed above. (Although see [51] for an off-shell BCJ relation for NLSM that is not of the form (8.3).)

**Strings and** ${\alpha ^{\prime }}$ **expansions.** The duality between ${\mathscr {L}}_n$ and ${\mathscr {L}}_n^*$ exploited in this paper is closely related to the geometry of ${{\mathcal {M}}}_{0,n}$, the moduli space of *n* points on the Riemann sphere. This is because the top-dimensional homology of ${{\mathcal {M}}}_{0,n}$ is generated by cycles naturally labelled by Lie polynomials; conversely, the top-dimensional cohomology is generated by cocycles naturally labelled by the elements of ${\mathscr {L}}_{n-1}^*$. This helps to explain the success of using ${{\mathcal {M}}}_{0,n}$ integrals, which arise in CHY formulas and in ambitwitor strings, to study tree level BCJ numerators for amplitudes. See for example [10, 52, 53], and details of the connection to ${\mathscr {L}}_n$ in [26]. This connection also strongly suggests an extension of our methods to string theory at tree level.

A perturbative method for computing the ${\alpha ^{\prime }}$ expansions of tree level string amplitudes using the ‘Drinfeld associator’ was given in [54]. The calculations reviewed in Sect. 7.6 can be related to this method by the equation of motion for $QV_P$ [55]. This equation of motion involves the contact term map, *C*, which is the dual of the *S*-bracket studied in this paper. This suggests that our results about the *S*-bracket will be useful for advancing the efforts to compute ${\alpha ^{\prime }}$ corrections in [50]. Moreover, the method in [54] is based on the Drinfeld associator, which is itself is a *Lie series*. This suggests that the duality between ${\mathscr {L}}$ and ${\mathscr {L}}^*$ will be central to advancing the use of this method to all orders in ${\alpha ^{\prime }}$.

**Beyond tree level.** It is an open question to discover whether KLT-like relations hold at higher loop order in the gauge theory perturbation series. As we have seen, tree level colour factors are labelled by Lie monomials, and partial tree amplitudes are labelled by permutations modulo shuffle relations. This is the leading order avatar of the more general story, at arbitrary orders in the perturbation series, in which colour factors are associated to ribbon graphs, and partial amplitudes are labelled by marked surfaces with boundary (possibly with genus $g>0$). The results in the present paper are essentially all derived from the Jacobi identity satisfied by Lie monomials is. Colour factors labelled by ribbon graphs at higher order satisfy analogous identities; as studied in [26]. This raises the possibility that the biadjoint scalar amplitudes at higher orders in perturbation theory play a role similar to role played by the ${\mathscr {L}}$-valued *b*(*P*) at tree level, in giving rise to KLT-relations.

## Appendix A. Berends–Giele Recursion for Yang–Mills in the Free Lie Algebra

Here we repeat the discussion of Sect. 3 replacing the biadjoint scalar by Yang-Mills. Consider pure Yang–Mills theory in *d*-dimensions with the Lagrangian

A.1 $$\begin{aligned} {{\mathcal {L}}}_{YM} = {1\over 4}\textrm{tr}({\mathbb {F}}_{\mu \nu }{\mathbb {F}}^{\mu \nu }),\qquad {\mathbb {F}}_{\mu \nu }:= -[\nabla _\mu , \nabla _\nu ]. \end{aligned}$$

The trace is over the generators $t^a$ of a Lie algebra, ${\mathfrak {g}}$, the covariant derivative is given by $\nabla _\mu = {\partial }_\mu - {\mathbb {A}}_\mu$ and ${\mathbb {A}}_\mu = {\mathbb {A}}_\mu ^a t^a$ is the gluon potential. In the Lorenz gauge, ${\partial }_\mu {\mathbb {A}}^\mu = 0$, the field equation $[\nabla _\mu , {\mathbb {F}}^{\mu \nu }] = 0$ becomes

A.2 $$\begin{aligned} \Box {\mathbb {A}}^\nu (x) = \left[ {\mathbb {A}}_\mu (x),{\partial }^\mu {\mathbb {A}}^\nu (x)\right] + \left[ {\mathbb {A}}_\mu (x),{\mathbb {F}}^{\mu \nu }(x)\right] . \end{aligned}$$

We now define a perturbative solution ${\mathbb {A}}$ that takes values in ${\mathscr {L}}$ rather than some given Lie algebra *L*. (A.2) leads to the following iteration for ${\mathbb {A}}_{\le n}$, with values in ${\mathscr {L}}_{\le n}$:

A.3 $$\begin{aligned} \begin{aligned} {\mathbb {A}}^\nu _{\le n+1}&={\mathbb {A}}_1^\nu + \textrm{proj}_{{\mathscr {L}}_{\le n+1}}\,\Box ^{-1} \left( [{\mathbb {A}}^\mu _{\le n}(x),{\partial }_\mu {\mathbb {A}}^\nu _{\le n}(x)] + [{\mathbb {A}}_{\le n\mu }(x),{\mathbb {F}}_{\le n}^{\mu \nu }(x)]\right) \,, \\{\mathbb {F}}^{\mu \nu }_{\le n+1}&=\textrm{proj}_{{\mathscr {L}}_{\le N+1}} \,2{\partial }^{[\mu }{\mathbb {A}}^{\nu ]}_{\le n+1}- [{\mathbb {A}}^\mu _{\le n},{\mathbb {A}}^\nu _{\le n}]. \end{aligned} \end{aligned}$$

where $\textrm{proj}_{{\mathscr {L}}_{\le n+1}}$ projects onto the ${\mathscr {L}}_{\le n+1}$ part. The recursion is seeded by

A.4 $$\begin{aligned} {\mathbb {A}}^\mu _1=\sum _{i\in {\mathbb {N}}} e_i^\mu \exp (ik_i\cdot x) \, i \end{aligned}$$

where the letter *i* replaces the usual generator $t^a_i\in {\mathfrak {g}}$ and $e_i^\mu$ is the polarization vector of the *i*th gluon. Note that $\Box ^{-1}$ acts on momentum eigenstates $\exp (ik\cdot x)$ to give $-1/k^2$.

To obtain the amplitude, define the multi-particle field ${\mathbb {A}}_{n-1}^\mu$, which is the degree $n-1$ part of ${\mathbb {A}}_{\le n-1}^\mu$:

A.5 $$\begin{aligned} {\mathbb {A}}_{n-1}^\mu :=\textrm{proj}_{{\mathscr {L}}_{n-1}} {\mathbb {A}}_{\le n-1}^\mu . \end{aligned}$$

In terms of ${\mathbb {A}}_{n-1}^\mu$, the ${\mathscr {L}}$-valued version of the amplitude is

A.6 $$\begin{aligned} \mathcal {A}_n= s_{12\ldots n-1}\,e^{-i k_{12\ldots n-1}\cdot x} \, {e_{n}}_\mu \,{\mathbb {A}}^\mu _{n-1} \,, \end{aligned}$$

where $e_n^\mu$ is the polarization of the *n*th gluon. The colour polarizations $t_1, \ldots , t_n\in {\mathfrak {g}}$ define the map $\textbf{t}:{\mathscr {L}}\rightarrow {\mathfrak {g}}$, and the YM amplitude is then given by $t_n^a\textbf{t}(\mathcal {A}_n)^a$. The partial tree amplitudes are given by

A.7 $$\begin{aligned} \mathcal {A}_n(P,n):=(P,\mathcal {A}_n), \end{aligned}$$

for permutations *P*.

The Berends–Giele current for an ordering *P* is

A.8 $$\begin{aligned} J^\mu _P:= (P, {\mathbb {A}}^\mu ) \exp (-ik_P\cdot x), \end{aligned}$$

where the factor of $\exp (-ik_P\cdot x)$ removes the *x*-dependence in $(P, {\mathbb {A}}^\mu _{|P|} )$. $J_P^\mu$ is linear in each $e_i$ with $i\in P$, with coefficients that depend only on the momenta. Taking inner products (A.3) with a word *P* gives the YM Berends–Giele recursion relation:

A.9 $$\begin{aligned} \begin{aligned} J^\mu _P&= {1\over s_{P}}\!\!\!\sum _{XY=P}\!\!\bigl [ J_{X}^\mu (k_X\cdot J_{Y}) + J_{X}^\nu {\mathcal {F}}_Y^{\mu \nu } - (X \leftrightarrow Y)\bigr ],\\{\mathcal {F}}^{\mu \nu }_Y&= k_Y^\mu J_Y^\nu - k_Y^\nu J_Y^\mu - \sum _{RS=Y}\big (J_R^\mu J_S^\nu - J_R^\nu J_S^\mu \big ) \end{aligned} \end{aligned}$$

where $J_i^\mu$ for a letter *i* is equal to the polarization vector $e^\mu _i$ of the *i*-th gluon. Here again we have used the following deconcatenation identity,

A.10 $$\begin{aligned} (P, \Gamma )=\sum _{XY=P} (X,\Gamma _1)(Y,\Gamma _2)-(X,\Gamma _2)(Y,\Gamma _1)\,, \end{aligned}$$

for a word *P*, and a Lie monomial $\Gamma = [\Gamma _1,\Gamma _2]$.

Since ${\mathbb {A}}_n\in {\mathscr {L}}$, it follows from Ree’s theorem that

A.11

cf. the discussions in [56] and [28]. In terms of $J^\mu _P$, the YM partial tree amplitudes are [31]

A.12 $$\begin{aligned} \mathcal {A}^\textrm{YM}(Pn) = s_{P}J_{P}\cdot J_n, \end{aligned}$$

which is equivalent to the earlier definition, (A.7).

## Appendix B. The Main Property of the *S*-Bracket

This appendix proves the following proposition, from § 4.2:

### Proposition

For $P,Q\in {\mathscr {L}}^*$, the *S* bracket $\{~,~\}$ satisfies

B.1 $$\begin{aligned} b(\{P,Q\}) = \left[ b(P),b(Q)\right] , \end{aligned}$$

i.e., *b* maps the *S*-bracket to the Lie bracket.

The proof uses the following definitions. The *deconcatenation coproduct* $\delta : W\rightarrow W\otimes W$ is defined on words *P* by

B.2 $$\begin{aligned} \delta (P)=\sum _{XY=P} X\otimes Y\,. \end{aligned}$$

Write $\delta '(P)$ to be as above but with the sum restricted to non-empty words *X*, *Y*. Further, write

B.3 $$\begin{aligned} \delta _\wedge (P)=\sum _{XY=P} X\otimes Y-Y\otimes X, \end{aligned}$$

and similarly for $\delta '_\wedge$. Finally, define the *S*-bracket to act on ${\mathscr {L}}^*\otimes {\mathscr {L}}^*$ as

B.4 $$\begin{aligned} \{X\otimes Y,Q\}=X\otimes \{Y,Q\}\,, \qquad \{P,X\otimes Y\}=\{ P,X\}\otimes Y\,. \end{aligned}$$

### Lemma

The deconcatenation of the *S*-bracket is

B.5 $$\begin{aligned} \delta '\{P,Q\}\sim \{\delta _\wedge P,Q\}+\{P,\delta _\wedge Q\}+ s_{P,Q} P\otimes Q\,. \end{aligned}$$

### Proof

First note that for non-empty *X*, *Y*

B.6 $$\begin{aligned} \delta '(XY)=\delta ' (X)\cdot ( e\otimes Y) +(X\otimes e)\cdot \delta ' (Y), +X\otimes Y \,, \end{aligned}$$

where *e* is the empty word. So

B.7

where we have used the explicit formulas for $\ell ^*$ and $r^*$ of (2.27). The KK relations give $XiY\sim i({\bar{X}}\,\, Y)\sim (X\,\, {\bar{Y}})i$, so the third term in (B.7) sums to $s_{P,Q}P\otimes Q$. The deconcatenation of $\ell ^*$ and $r^*$ can be evaluated using (2.27). For example, the deconcatenation of a single term in (2.27) is

B.8

Total shuffles vanish in ${\mathscr {L}}^*$. So, in ${\mathscr {L}}^* \otimes {\mathscr {L}}^*$,

B.9

This can be used to find that

B.10 $$\begin{aligned} \delta ' (\ell ^*(P))= (\ell ^*\otimes 1) \circ \delta '_\wedge (P), \end{aligned}$$

and a similar identity for $r^*(Q)$. $\square$

### Proof

(of the proposition) When *P*, *Q* are single letters, (B.1) follows directly. Note that BG recursion can be written as

B.11 $$\begin{aligned} b(P)=\frac{1}{s_P} \sum _{X,Y} (X\otimes Y,\delta '(P))\, [b(X),b(Y)], \end{aligned}$$

for any homogeneous $P \in {\mathscr {L}}^*$. Substituting $\{P,Q\}$ for *P* into this recursion, the lemma gives that

B.12 $$\begin{aligned} \begin{aligned} s_{PQ} b(\{P,Q\})&= \sum _{X,Y} \left( X\otimes Y, \{\delta '_\wedge P,Q\}+\{P,\delta '_\wedge (Q)\}+s_{P,Q} P\otimes Q\right) \, \left[ b(X),b(Y)\right] \,, \\&= s_{P,Q} \left[ b(P),b(Q)\right] \\&\qquad +\sum _{P=XY} \left[ b(X), b(\{Y,Q\})\right] - (X\leftrightarrow Y) \\&\qquad + \sum _{Q=XY} \left[ b(\{P,X\}), b(Y)\right] - (X\leftrightarrow Y) \,. \end{aligned} \end{aligned}$$

By induction, write $b(\{Y,Q\})=[b(Y),B(Q)]$ in the second last line to find

B.13 $$\begin{aligned}{} & {} \sum _{P=XY} [b(X), [b(Y),b(Q)] - (X\leftrightarrow Y)\nonumber \\{} & {} =\sum _{P=XY}[[b(X),b(Y)],b(Q)] = s_P[b(P),b(Q)]\,, \end{aligned}$$

using the Jacobi identity followed by BG recursion. The same argument applied to the third line shows that the RHS of (B.12) is [*b*(*P*), *b*(*Q*)] multiplied by $s_{PQ}$. $\square$

## Appendix C. The KLT Matrix Elements

This appendix recovers again the standard formula for the KLT matrix,

C.1 $$\begin{aligned} S^\ell (1A|1B)=\prod _{i=2}^ns_{i,A_i(A|B)}, \end{aligned}$$

where $A_i(A|B)$ is the subset of $\{1,2,3,\ldots ,n\}$ containing all letters that both precede *i* in 1*B* and follow *i* in 1*A*. We explicitly compute $S^\ell (12\ldots n|1A)$ by expanding $\ell [12\ldots n]$, which is in spirit similar to the argument in [16]. The idea is to iteratively apply our main identity:

C.2 $$\begin{aligned} \left[ b(P),k\right] = b(\{P,k\}), \end{aligned}$$

for a letter *k*. Using the formula for $\{P,k\}$, equation (4.12), this gives

C.3 $$\begin{aligned} \begin{aligned} \left[ \ldots [[1,2]3]\ldots ,n\right]&= s_{12}[\ldots [b(12),3]\ldots ,n]\\&= s_{12}s_{1,3} [\ldots [b(132),4]\ldots ,n]\\&\qquad + s_{12} s_{12,3}[\ldots [b(123),4]\ldots ,n]\\&=\ldots \end{aligned} \end{aligned}$$

The RHS can be resummed to give the nested sum:

C.4 $$\begin{aligned} RHS = s_{12}\left( \sum _{12=A_2B_2} s_{2,A_2} \left( \ldots \left( \sum _{A_{n-1}nB_{n-1}-A_nB_n} s_{n,A_n} b(A_nnB_n)\right) \ldots \right) \right) .\qquad \end{aligned}$$

Here all the words $A_i$ begin with the letter 1 and by convention $s_{i,A}=0$ when *A* is empty. $S^\ell (12\ldots n| 1A)$ is the coefficient of *b*(1*A*) in (C.4). Reversing the order of the summations gives this coefficient as

C.5 $$\begin{aligned} S^\ell (12\ldots n| 1A)= \prod _{i=2}^n \left( \sum _{C_i=A_iiB_i} s_{i,A_i}\right) \end{aligned}$$

where $C_i$ is the word 1*A* with the letters $1,\ldots ,i-1$ removed. In other words, $A_i$ is a word in the letters *j* such that $j<i$ in the ordering 1*A* and $i<j$ in the ordering $12\ldots n$.

## Footnotes

1 The colour factor $c_\Gamma$ is formed from the graph $\Gamma$ by associating Lie algebra structure constants at each vertex, Kronecker-deltas along each internal propagator and taking inner products with the ‘external colours’ at the leaves.

2 We will describe the space of functions of kinematics that arise in amplitudes in general. But it is possible to describe the image of this homomorphism for any gauge theory that participates in the double copy.

3 Another way to find (2.20) is to prove the following identity at the level of words:  This is stated in [28] and proven in [29] (see also equation (41) in [30]).

4 In [14], the perturbative solution to the biadjoint scalar field equations was also written as a perturbiner-like [32] expansion in the spirit of Berends–Giele [31] leading to the desired recursion (3.10) for *b*(*P*) that we find below. Also, the identity  was proved using different methods in the appendix of [28].

5 Alternative formulas for the generalized KLT matrix arise by choosing any pair of dual bases. E.g. one can define $S^{r} (P,Q):= (r\{P\},r(Q))$, but as $P, Q \in {\mathscr {L}}$, by Lemma 1.7 of [1], $r\{P\}:=-\ell \{\alpha (P)\}$ and $r(P)=-\ell (\alpha (P))$ so $S^{r}(P|Q)=S^{\ell }({\bar{P}}|{\bar{Q}})$. These alternative choices explain redefinitions during its evolution to the version given in [40], see e.g. footnote 10 in [41]. As pointed out in [13], the generalized KLT matrix $S^\ell (P|Q)$ can be defined for *any* two words *P*, *Q*, instead of restricting *P*, *Q* to a set of Lyndon words. This was proposed in [13] to eliminate the choice of special leg *i* in the standard definition of the KLT matrix $S(R|S)_i$. This prevented the KLT matrix $S(R|S)_i$ from satisfying generalized Jacobi identities in *R* and *S* given in (5.40).

6 The identity, (5.40), motivated the introduction of the generalized KLT matrix in [13]. In the multiparticle pure spinor superfield framework, where $V_P$ are local superfields satisfying generalized Jacobi identities and $M_Q$ are Berends–Giele current superfields satisfying shuffle symmetries, we have the relation $V_{iA} = \sum _B S(A|B)_i M_B$. However, the fixed leg *i* prevents the Jacobi identities of $V_{iA}$ being manifest. See § 7.5 for further discussion.

7 Their approach was to extract the numerators as the coefficients of $1/s_\Gamma$ and to show that these $N_\Gamma$ could not be chosen to be BCJ. This led the authors to the introduction of ‘BCJ gauge’ in which local numerators are obtained.

8 An arbitrary set of local on-shell BCJ numerators for the theory may not be the restriction of the off-shell numerators, because of the gauge freedom, (7.11).
